# CD146 expression profile in human skin and pre-vascularized dermo-epidermal skin substitutes in vivo

**DOI:** 10.1186/s13036-023-00327-x

**Published:** 2023-01-31

**Authors:** Monica Nanni, Dominic Rütsche, Curdin Bächler, Luca Pontiggia, Agnes S. Klar, Ueli Moehrlen, Thomas Biedermann

**Affiliations:** 1 Tissue Biology Research Unit, Department of Surgery, University Children’s Hospital Zurich, Wagistrasse 12, 8952 Zurich, Switzerland; 2 Children’s Research Center, University Children’s Hospital Zurich, Zurich, Switzerland; 3 Department of Mechanical and Process Engineering, Institute for Mechanical Systems, ETH Zurich, Leonhardstrasse 21, 8092 Zurich, Switzerland; 4 Department of Surgery, University Children’s Hospital Zurich, Zurich, Switzerland; 5 University of Zurich, Zurich, Switzerland

**Keywords:** CD146, Blood capillaries, Pericytes, 3D vascular network, Tissue engineering, Inflammation

## Abstract

**Background:**

CD146 is a cell adhesion molecule whose expression profile in human skin has not yet been elucidated. Here, we characterize CD146 expression pattern in human skin, in particular in blood endothelial cells (BECs) and lymphatic endothelial cells (LECs), which constitute human dermal microvascular endothelial cells (HDMECs), as well as in perivascular cells.

**Results:**

We demonstrated that CD146 is a specific marker of BECs, but not of LECs. Moreover, we found CD146 expression also in human pericytes surrounding blood capillaries in human skin. In addition, we demonstrated that CD146 expression is up-regulated by the TNFα-IL-1β/NF-kB axis in both BECs and pericytes. Finally, we engineered 3D collagen hydrogels composed of HDMECs, CD146^+^ pericytes, and fibroblasts which developed, in vitro and in vivo, a complete microvasculature network composed of blood and lymphatic capillaries with pericytes investing blood capillaries.

**Conclusions:**

Overall, our results proved that CD146 is a specific marker of BECs and pericytes, but not LECs in human skin. Further, the combination of CD146^+^ pericytes with HDMECs in skin substitutes allowed to bioengineer a comprehensive 3D in vitro and in vivo model of the human dermal microvasculature.

**Supplementary Information:**

The online version contains supplementary material available at 10.1186/s13036-023-00327-x.

## Introduction

CD146, also known as melanoma cell adhesion molecule (MCAM), hemopoietic cell adhesion molecule (HEMCAM), MUC18, S-Endo1, or A32 antigen, is a cell surface glycoprotein belonging to the immunoglobulin superfamily. CD146 is expressed in the entire vascular tree, independently from vessel caliber or anatomical location (1, 2). Alternative splicing of the exon 15 generates two transmembrane isoforms of CD146, long (lgCD146) and short (shCD146), while a third soluble isoform of CD146 (sCD146) is generated by cleavage of the former [1, 2]. sCD146 is found in the serum and interstitial fluids of both healthy and pathological subjects, and is involved in vessel formation, cell migration, and inflammation [3, 4].

CD146 is constitutively expressed in the intercellular junction of endothelial cells, where it regulates cell adhesion, vessel integrity, and permeability [5]. In addition, CD146 is also involved in migration, proliferation, angiogenesis, and ﻿trans-endothelial migration [1]. Originally, CD146 has also been described originally as a marker of melanoma growth and metastasis [6], and was found aberrantly expressed in several vascular disorders [7–9].

A recent study revealed that CD146 is also expressed in pericytes of different organs like skeletal muscle, pancreas, adipose tissue, and placenta, where it stabilizes capillaries and small venules [10]. In particular, CD146 has been well characterized in the ﻿blood–brain barrier (BBB), where it is involved in blood–brain-barrier maturation and stabilization, coordinating pericyte-endothelial cell communication [11], and regulating PDGF-B/PDGFRβ-induced pericyte recruitment ﻿to vessels of the central nervous system [12].

Despite the well-recognized role of CD146 in angiogenesis regulation, CD146 also plays an important role during inflammation. In fact, CD146 expression can be modulated in an inflammatory environment following exposition with different stimuli, such as growth factors, hypoxia, osmotic pressure and proinflammatory cytokines [13, 14]. In particular, CD146 expression can be up-regulated after stimulation with TNFα and IL-1β through ﻿NF-kB transactivation in human umbilical vein endothelial cells (HUVECs) [15, 16]. However, to date no data is available on the effect of TNFα and IL-1β stimulation on pericytes.

﻿ The expression profile of CD146 in human skin has been described only in a few studies, which mainly involved endothelial cells [17, 18]. Meanwhile, its characterization in human dermal pericytes is missing. Moreover, there is conflict evidence regarding CD146 expression in blood and lymphatic endothelial cells. Indeed, while gene expression analysis on these cells identified CD146 as specific marker of blood endothelial cells [18], another study showed that CD146 is expressed in lymphatic endothelial cells and exerts a critical role in VEGF-C mediated lymphangiogenesis [19].

Here, we report a profound study of CD146 expression in human skin. Our data conclusively characterize CD146 as a specific marker of blood endothelial cells, but not lymphatic endothelial cells. In addition, we show that CD146 is a specific marker of human dermal pericytes, extensively expressed throughout the human dermal microvasculature. We also demonstrate that the stimulation with the pro-inflammatory cytokines, TNFα and IL-1β, induces CD146 up-regulation in vitro in both pericytes and BECs, via NF-kB activation. Finally, we engineer pre-vascularized skin substitutes containing CD146^+^ pericytes combined with human dermal microvascular endothelial cells (HDMECs) and fibroblasts, in order to obtain a microvascular network that closely resembles the human dermal microvasculature in vitro and in vivo.

## Materials and methods

### Human skin samples

Human foreskin samples were obtained from patients between 1–10 years of age after their parents gave written informed consent. Tissue samples for histological examination were embedded in OCT compound (Sakura Finetek, Switzerland) and stored at − 20 °C. Alternatively, biopsies were singularly used for cell isolation.

### Cell isolation and culture

HDMECs were positively isolated from skin tissue digests with Dynabeads® CD31 (Thermo Fisher Scientific, Basel, Switzerland), according to the manufacturer’s instructions. Briefly, tissues were minced into small pieces and digested in 0,5% collagenase type II (Worthington, Lakewood, NJ) diluted in Dulbecco’s Modified Eagle Medium (DMEM, GIBCO, Thermo Fisher Scientific) plus 1% penicillin–streptomycin (GIBCO) for 40 min at 37 °C. Thereafter, the reaction was stopped adding DMEM^+++^ (DMEM supplemented with 10% fetal calf serum (GIBCO), 1% HEPES (GIBCO), and 1% penicillin–streptomycin). The cell suspension was filtered through a 100 μm cell strainer and centrifuged 5 min at 200 g. Cells were incubated with anti-CD31 coated Dynabeads for 20 min at 2–8 °C with gentle rotation. Afterward, the bead-bound cells were separated by a magnet and the CD31^−^ fraction was collected in a separated tube. CD31^+^ HDMECs were washed 3 times with a buffer solution (0.1% of bovine serum albumin (BSA) in phosphate-buffered saline (PBS)) and separated using a magnet. Finally, the bead-bound HDMECs were resuspended in EGM-2MV medium (Lonza, Basel, Switzerland) and seeded on 5 cm dishes pre-coated with collagen (0.1 mg/ml, Symatese, Chaponost, France). CD146^+^ pericytes were isolated from the previously described CD31^−^ fraction with CELLection biotin binder magnetic beads (Thermo Fisher Scientific) according to the manufacturer’s instructions. Briefly, cells were incubated with biotinylated anti-CD146 antibody (BioLegend, Lucerne, Switzerland) for 10 min at 2–8 °C, followed by incubation with anti-biotin beads for 20 min at 2–8 °C with gentle rotation. Thereafter, the bead-bound cells were separated and washed by a magnet as reported above. Finally, CD146^+^ pericytes were resuspended in DMEM^+++^ and plated on 5 cm dishes. HDMECs and CD146^+^ pericyte characterization was assessed by immunofluorescence and western blot.

Human dermal fibroblasts (FBs) and human keratinocytes (KCs) were isolated and cultured as previously described [20].

### Cell treatment

BECs and CD146^+^ pericytes isolated from 3 independent biological donors were seeded into 6 well plates and cultured with EGM-2MV or DMEM^+++^ respectively. Cells ﻿were used at passage 2 and grown to 70% of confluence and then treated with human Tumor-Necrosis-Factor-alpha (TNF-α, 50 ng/ml, Peprotech, Germany) or IL-1β (10 ng/ml, Peprotech) or IL-6 (20 ng/ml, Peprotech) for either 6 h, 12 h, or 24 h. The treatment was stopped at the indicated time point and cells used for western blot analysis. To inhibit NF-κB pathway, cells were pre-treated with BAY 11–7082 (5 μM; Sigma, Buchs, Switzerland) for 1 h at 37 °C before being treated with TNF-α or IL-1β in the presence of the inhibitor.

### Cell viability assay

Cell viability assay was assessed by fluorescein diacetate–propodium iodide (FdA–PI) live staining. Cells isolated from 3 independent biological donors were used at passage 2 (P2). Briefly, cells were washed in PBS and then incubated with a solution of FdA (80 µg/mL; Sigma) and PI (200 µg/mL; Sigma), for 2 min at 37 °C. Then cells were washed twice in PBS before applying the fresh medium. Fluorescence was immediately checked using an inverted Microscope (Nikon Eclipse TE2000-U) connected with DXM1200F digital camera.

### Pre-vascularized dermo-epidermal skin substitute production

Pre-vascularized collagen hydrogels were prepared as described previously [21]. Briefly, cultured HDMECs, pericytes and fibroblasts were harvested at passage 0 and resuspended in 2 ml collagen type I (Symatese) in a ratio of 2:1:1 (150.000 cells/ml in total). The mixture was immediately cast in 6-well cell culture inserts (3.0 µm pore-size membranes, BD Falcon, Basel, Switzerland) and cultured with EGM-2MV medium (Lonza) for three weeks. Subsequently, 1 × 10^6 ^keratinocytes (passage 0) were distributed onto each dermal equivalent. The skin equivalents were cultured with EGM-2MV in the lower chamber and CnT57 ﻿(CellnTec, Bern, Switzerland) in the upper chamber for 1 week and subsequently transplanted. Three different skin cell donors (*n* = 3) were used for hydrogel preparation.

### Transplantation of tissue-engineered skin substitutes

Pre-vascularized dermo-epidermal skin substitutes were transplanted onto full-thickness skin defects created surgically on the back of 10-week-old female, athymic Nu/Nu immune-compromised rats (Envigo, Horst, The Netherlands) (*n* = 3). Animals were anesthetized by inhalation of 5% Isoflurane (Baxter, Volketswil, Switzerland) and maintained by inhalation of 2.5% Isoflurane via mask. Full-thickness skin wounds were created in the middle of the back of the animals with a surgical scissor. A custom-made steel ring (diameter 26 mm) was then placed into the created full-thickness skin defect and the skin was sutured using non-absorbable polyester sutures (Ethibond®, Ethicon, Raritan, NJ, USA), on the outside of the ring to prevent wound closure by the rat skin. The skin substitutes were placed into the metal ring onto the animal tissue. The transplants were then subsequentially covered with a silicone foil (Silon-SES, BMS, New York, NY, USA), a polyurethane sponge (Ligasano, Ligamed, Innsbruck, Austria), a cohesive conforming bandage (Sincohaft, Theo Frey AG, Bern, Switzerland), and tape as a wound dressing. After 1 week animals were sacrificed and transplants were excised and processed for histological and immunofluorescence analysis.

### Histological analysis

Paraffin sections (5 μm) were stained with hematoxylin and eosin (Sigma) to assess the histological morphology of the skin grafts*.*

### Immunofluorescence analysis

For immunocytochemistry, cells were grown on coverslips and then fixed in ice cold acetone/methanol (1:1) for 5 min at -20 °C. For immunofluorescence staining (IF) of cryosections, cryoembedded skin biopsies were cut with Leica CM3050S cryostat (10 μm thickness) and placed on SuperFrost Plus glasses (Thermo Fischer Scientific). Sections were rehydrated and washed three times with PBS, fixed and permeabilized on ice cold acetone/methanol (1:1) for 5 min at -20 °C. After fixation cells/cryosections were dried for 30 s and blocked for 30 min at room temperature (RT) with blocking buffer (2% of BSA in PBS). The primary antibodies diluted in blocking buffer were incubated for 1 h at RT. Cells and/or cryosections were washed three times with PBS and incubated with secondary antibodies diluted in blocking buffer for 30 min at RT. When necessary, the same procedure was repeated for a third antibody incubation, with additional 15 min of blocking in between. Finally, cells and/or sections were washed in PBS and mounted with Dako mounting solution (Dako, Baar, Switzerland) containing 25 mg/ml of DABCO anti-quenching agent (Sigma). The paraffin sections from transplanted skin grafts were deparaffinized in xylol and rehydrated through graded ethanol dilutions to PBS. Antigen retrieval was performed by incubating sections with protease XXIV (0.1 mg/ml) for 20 min at RT. Sections were then blocked with 10% BSA in PBS for 30 min at RT (Sigma), before incubation with the primary antibody diluted in blocking buffer overnight at 4 °C. Sections were washed three times with PBS and incubated with secondary antibodies diluted in blocking buffer for 30 min at RT. Sections were washed in PBS and mounted with Dako mounting solution (Dako) containing 25 mg/ml of DABCO anti-quenching agent (Sigma). For whole-mount immunostainings, hydrogels were fixed in neutralized 4% paraformaldehyde (PFA; J.T. Baker) for 4 h at 4 °C and washed for at least 6 h with PBS, changing the solution every 30 min. Thereafter, the samples were permeabilized with 0.3% Triton X-100 (Sigma) in PBS for 1 h at 4 °C and blocked for 2 h at 4 °C with the immunomix (PBS, 10% BSA, 0.05% sodium azide). Samples were incubated with primary antibodies diluted in immunomix overnight at 4 °C. The hydrogels were washed with PBS for 12 h and incubated with secondary antibodies diluted in immunomix overnight at 4 °C. The images were taken with an inverse confocal laser scanning microscope (Leica SP8, Heerbrugg, Switzerland) or an inverted Microscope (Nikon Eclipse TE2000-U) connected with DXM1200F digital camera. Antibody suppliers, catalog numbers and working dilution are shown in Supplementary Table 1.

### Flow cytometry

Freshly isolated cells from four independent skin samples were analyzed by flow cytometry to characterize HDMEC and pericyte populations. After collagenase digestion, the cell suspension was incubated for 30 min at 4 °C with primary antibodies or isotype-matched control antibodies diluted in FACS buffer (0.5% of BSA in PBS). Zombie Aqua (live/dead dye) (1:500, BioLegend) was used to exclude dead cells. After incubation, cells were washed twice with FACS Buffer and centrifuged at 200 g for 5’ at 4 °C. Pellet was resuspended in FACS Buffer and filtered through a tube with a cell strainer cap. For FACS analysis cells were fixed with 4% PFA for 5 min following an additional washing step and analyzed with BD LSRFortessa flow cytometer (BD Biosciences, Allschwil, Switzerland). Alternatively, not fixed cells were sorted using the BD FACSAriaTM III FACS sorter (BD Biosciences). For gate setting and compensation, unlabelled, single-labeled cells and fluorescence minus one (FMO) were used as control. Data analysis was performed using FlowJo (BD Biosciences). Antibody suppliers, catalog numbers and working dilution are shown in Supplementary Table 1.

### Western blot

The cells were washed with ice-cold PBS twice and lysed in Ripa buffer (Sigma) containing complete protease C (Roche, Basel, Switzerland) and phosphatase inhibitors (1 mM sodium orthovanadate, 20 mM sodium pyrophosphatase, 50 mM sodium fluoride, all from Sigma). The cell lysates were centrifuged at 16.000 g at 4 °C for 20 min and supernatants were collected. The protein concentrations were determined by Bio-Rad protein assay reagent. The samples were denatured by adding Laemmli Buffer (Biorad, Cressier, Switzerland) at 95 °C for 5 min. A range between 10 and 20 μg of total protein was analyzed by SDS–polyacrylamide gel electrophoresis using pre-casted gels (Biorad) and transferred to nitrocellulose membranes (Biorad) using Trans-Blot Turbo transfer system (BioRad). The membranes were blocked for 1 h at RT with 3% non-fat dry milk (Biorad) or with 3% BSA in TBST (PBS plus 0,1% Tween-20). The blots were incubated with the indicated antibodies in blocking solution overnight at 4 °C. Afterwards, the membranes were washed three times with TBST followed by incubation with the appropriate secondary horseradish peroxidase (HRP)-labeled antibodies (Dako) for 1 h at RT. The membranes were washed three times and results were detected with Clarity™ Western ECL Substrate (Biorad) and the signal acquired by Syngene G-Box (Syngene, United Kingdom). Densitometric analysis was performed using Image J. Antibody suppliers, catalog numbers and working dilution are shown in Supplementary Table 1.

### Statistical analysis

All the data are reported as mean ± standard deviation (SD). The statistical analyses were performed with GraphPad Prism 4.0 (Graph Pad Software, La Jolla, CA, USA). Comparison between the two groups was performed using the unpaired Student’s t-test. Significance was determined for *P* < 0.05.

## Results

### CD146 is expressed on blood capillaries in normal human skin

**Fig. 1:**
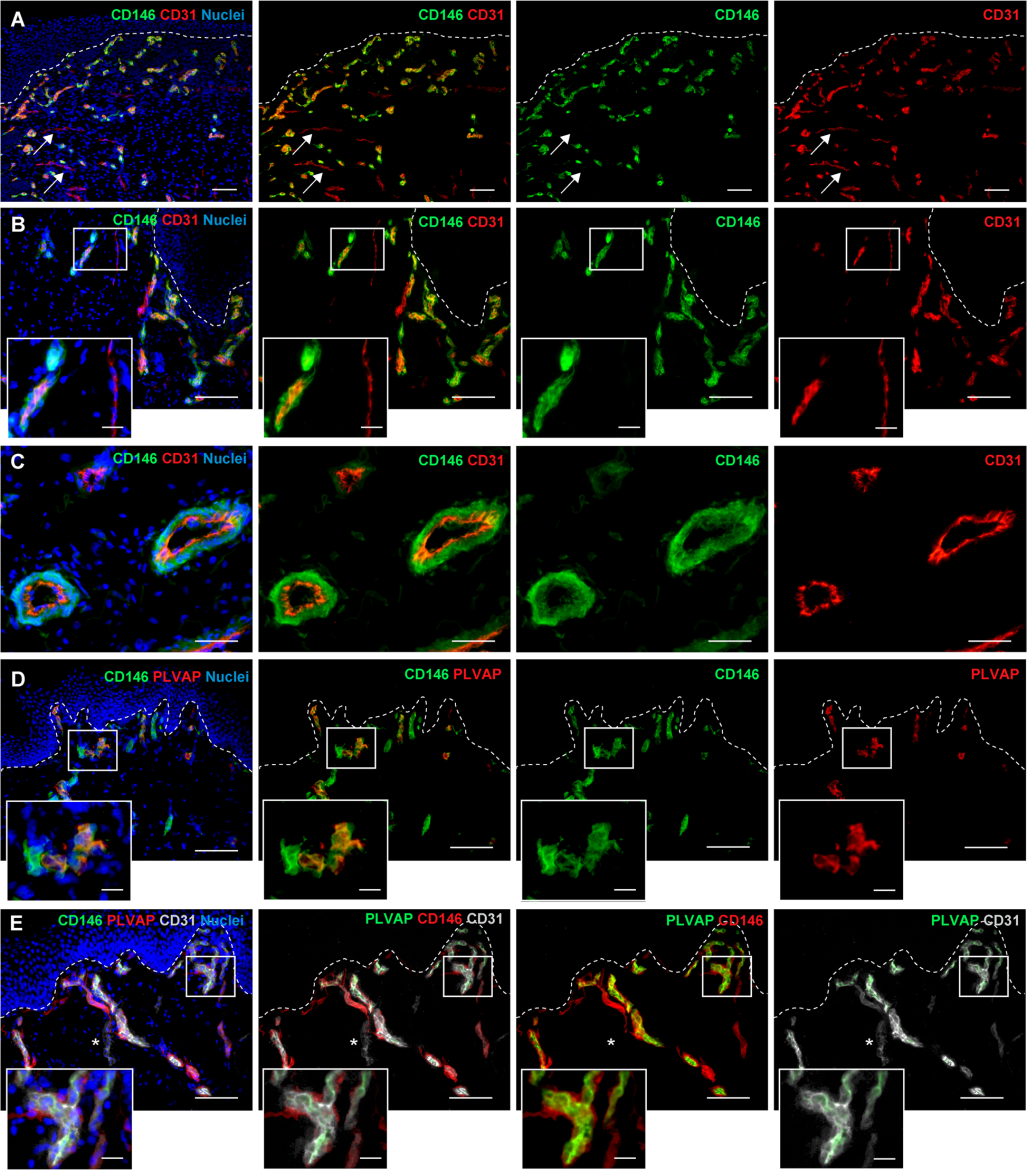
CD146 expression in normal human skin.** A**,** B**, **C** Representative immunofluorescence images of CD146 and CD31 of normal human skin. Co-localization between CD146 and CD31 is observed on the majority of capillaries, in both upper and lower dermis. A small fraction of CD31 positive capillaries results negative for CD146 (arrow and inset). Single positive cells for CD146 are observed around CD31 positive endothelial cells. Scale bar: **A**, **B**: 100 µm, **C**: 50 µm, inset: 20 µm. **D** Double immunofluorescence analysis of CD146 and PLVAP shows that CD146 co-localizes with PLVAP-positive endothelial cells and is expressed in the surrounding perivascular cells. Scale bar: 50 µm. **E** Triple immunofluorescence staining for CD146, PLVAP and CD31 confirms the co-localization between CD146, PLVAP and CD31. A fraction of CD31^+^CD146^−^PLVAP^−^ is observed, probably corresponding to lymphatic capillaries (asterisk). (*n* = 3 independent donors). Scale bar: 50 µm. White dashed lines indicate the epidermal-dermal junction

To investigate the distribution of CD146 on the dermal microvasculature of normal human skin in situ, we analyzed its expression by double immunofluorescence staining with two specific endothelial markers, CD31 or Plasmalemma vesicle-associated protein (PLVAP, also known as PAL-E, PV-1 and FELS) (Fig. 1). We observed co-localization of CD146 with the majority of CD31 positive capillaries (Fig. 1A-C), while only a small fraction of CD31 positive capillaries were negative for CD146 (Fig. 1A, white arrow, Fig. 1B, inset). A high density of CD146/CD31 double positive capillaries was detected in the superficial capillary plexus of the papillary dermis (Fig. 1A, B), although CD146 was also observed in large vessels of the deep plexus of the reticular dermis (Fig. 1C). In addition, CD146 positive cells were found around CD31 positive endothelial cells surrounding the capillary wall (Fig. 1A-C), indicating that CD146 was expressed in both endothelial and perivascular cells in human skin.

**Fig. 2:**
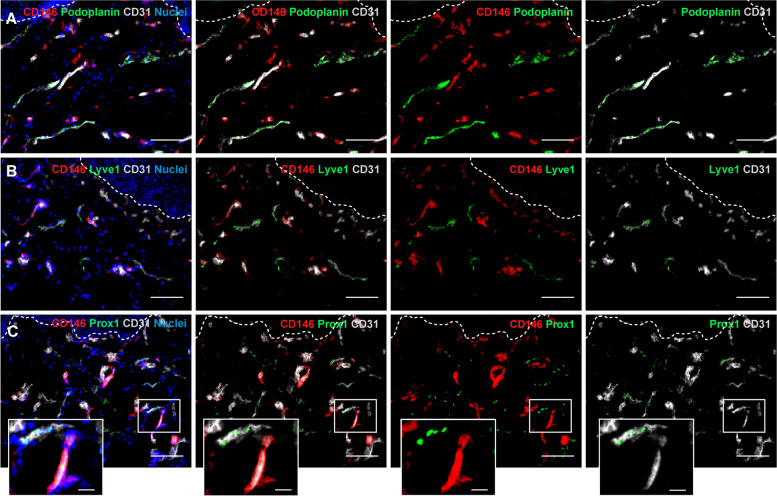
Lymphatic capillaries are negative for CD146 expression. **A**, **B**, **C** Lymphatic and blood capillaries are discriminated as CD31 and Podoplanin/Lyve1/Prox1 double positive and CD31 positive Podoplanin/Lyve1/Prox1 negative, respectively. Representative immunofluorescence images show no expression of CD146 on lymphatic capillaries positive for Lyve1 (**A**), Podoplanin (**B**) and Prox1 (**C**, inset). CD146 expression is detected only on blood capillaries and their related perivascular cells. (*n* = 3 independent donors). Scale bar: 50 µm. White dashed lines indicate the epidermal-dermal junction

The observed small fraction of CD146-negative capillaries (Fig. 1A, arrows, Fig. 1B, inset) suggested that this adhesion molecule is not uniformly expressed in the total skin microcirculation. Since CD31 does not discriminate between blood and lymphatic capillaries, we investigated the expression of CD146 using the specific blood vessel marker PLVAP [22, 23]. First, we confirmed the expression of PLVAP on blood capillaries, which is mutually exclusive with that of the lymphatic capillary markers, Podoplanin, Lyve1 and Prox1 (Supplementary Fig. S1A-D). Indeed, these markers can be interchangeable to discriminate and/or isolate lymphatic endothelial cells (LECs) [18, 24]. We showed that CD146 localized in both PLVAP-positive endothelial cells and surrounding perivascular cells (Fig. 1D, inset). Triple immunofluorescence staining confirmed the co-expression of CD146 with CD31 and PLVAP (Fig. 1E) and highlighted CD31^+^CD146^−^PLVAP^−^ cells corresponding to lymphatic capillaries (Fig. 1E, asterisk). The individual channels of each staining are reported in Supplementary Fig. S1E.

Further, we definitely excluded the expression of CD146 on lymphatic skin capillaries. Lymphatic and blood capillaries were identified as CD31 and Podoplanin/Lyve1/Prox1 double-positive and CD31 positive Podoplanin/Lyve1/Prox1 negative, respectively. All the lymphatic capillaries alternatively stained for Podoplanin, Lyve1 or Prox1 showed no CD146 expression, which was instead evident only in blood capillaries and in their surrounding perivascular cells (Fig. 2A-C, inset). The single channels are reported in Supplementary Fig. S2.

### CD146 is expressed on pericytes in normal human skin

**Fig. 3:**
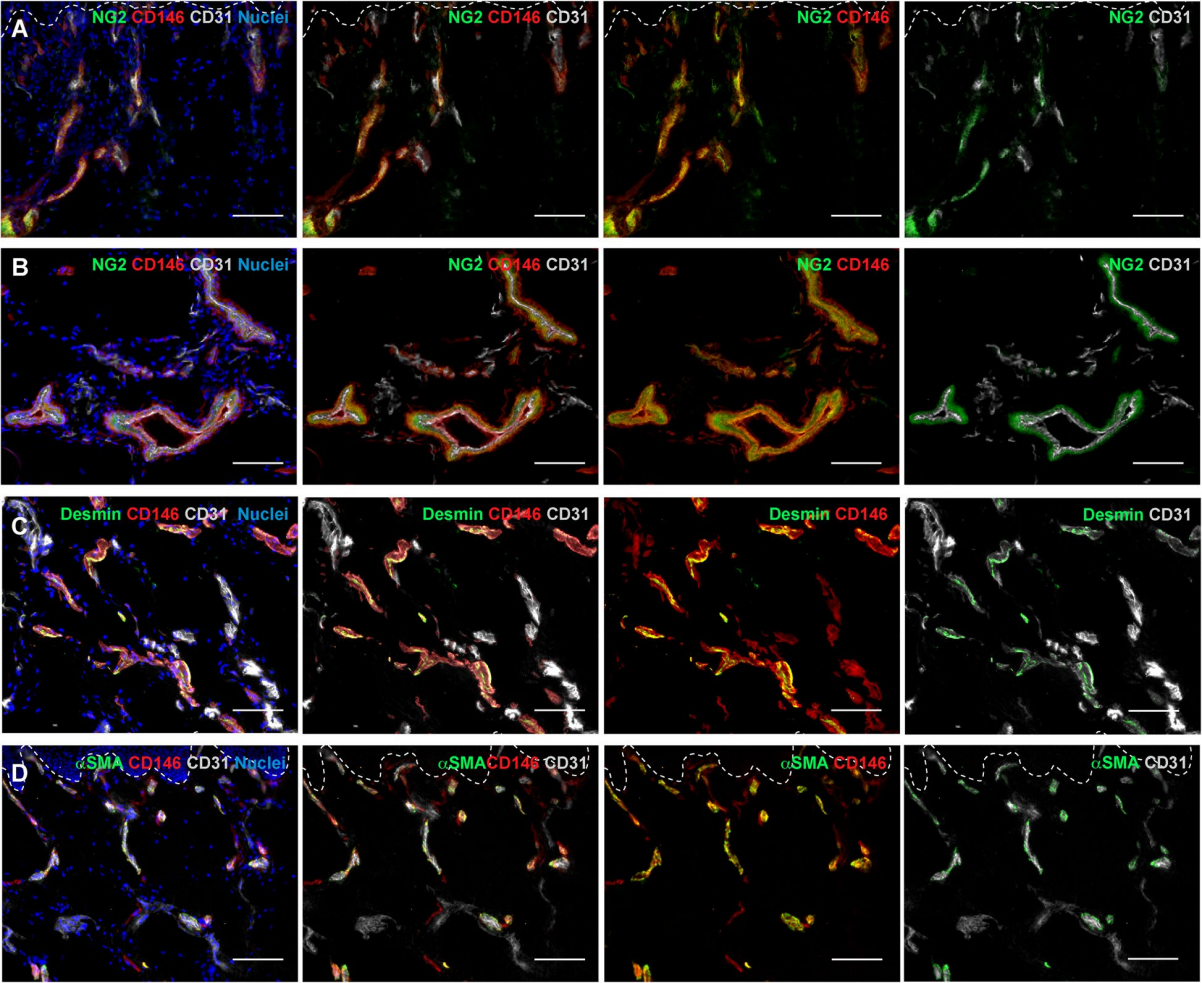
CD146 expression on pericytes in normal human skin. **A**, **B**, **C**, **D** Representative immunofluorescence images of human skin sections stained for CD146, CD31 and different markers of perivascular cells. Pericyte-like cells double positive for CD146 and NG2 localize around the entire blood capillaries, in both upper (**A**) and deep (**B**) vascular plexus. NG2^−^/CD146^+^/CD31^+^ blood endothelial cells and NG2^+^/CD146^+^/CD31^−^ pericytes can be clearly distinguished in blood capillaries (**B**). Whereas a small population of desmin/CD146-double positive cells can be observed only on a small fraction of blood capillaries (**C**), co-localization between CD146 and αSMA is extensively observed in most of the blood capillaries (**D**). (*n* = 3 independent donors). Scale bar: 50 µm. White dashed lines indicate the epidermal-dermal junction

To ascertain that the single positive cells for CD146 detected in close proximity to CD31^+^ blood capillaries correspond to pericytes, we performed triple immunofluorescence co-staining for CD146, CD31, and different specific markers of perivascular cells: NG2, desmin, and αSMA [10] (Fig. 3). A clear co-localization between CD146 and NG2 was observed all around CD31 positive blood capillaries, where they seemed to continuously invest the capillary wall (Fig. 3A). CD146 and NG2 appeared to be ubiquitously expressed on all perivascular cells of the blood capillaries both in the upper superficial capillary plexus (Fig. 3A) and deep vascular plexus of the reticular dermis (Fig. 3B). On the other hand, a co-localization between desmin and CD146 was observed only in the pericytes of the lower dermis (Fig. 3C), while desmin expression was almost undetectable in the upper dermis (data not shown). Among the well-known markers of perivascular cells, αSMA is mostly expressed around venules and arterioles, but is usually not detected around capillaries [10, 25]. We observed a co-localization between CD146 and αSMA (Fig. 3D) indicating that CD146 is expressed in perivascular cells around capillaries, venules and arterioles. The single channels are reported in Supplementary Fig. S3.

### CD146 is expressed on blood endothelial cells in vitro

**Fig. 4:**
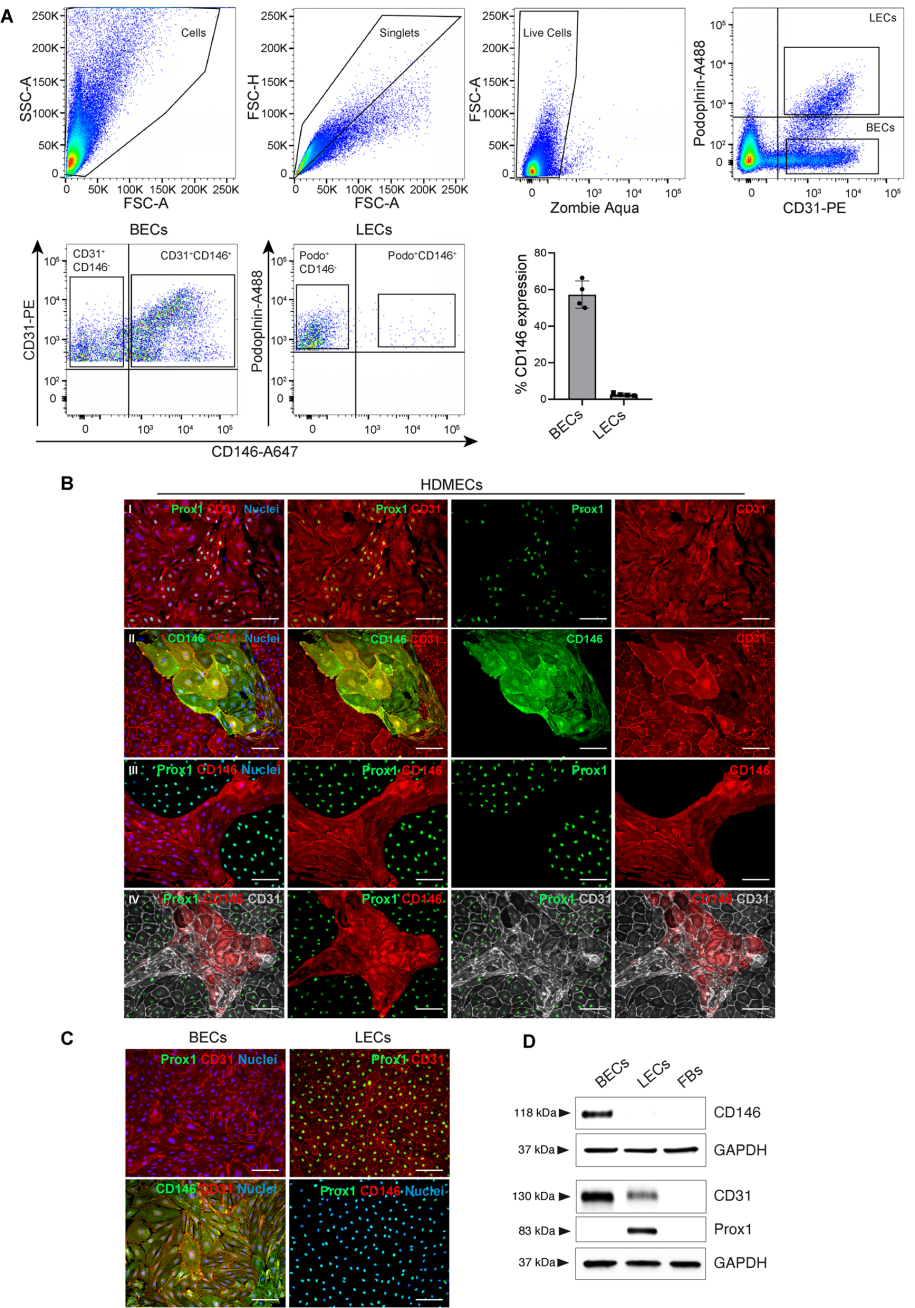
Expression of CD146 on cultured HDMECs and sorted BECs and LECs. **A** Flow cytometry analysis of freshly isolated HDMECs from human foreskin dermis. The hierarchical gating strategy involved the consecutive exclusion of debris, doublets and dead cells using Zombie Aqua staining. CD31 and Podoplanin were used to discriminate between BECs (CD31^+^Podoplanin^−^) and LECs (CD31^+^Podoplanin^+^). CD146 expression was detected only on freshly isolated BECs (57,3 ± 7,4%), while freshly isolated LECs were almost completely negative for CD146 expression (2,6 ± 0,7%). ﻿*n* = 4 independent skin donors. **B** In vitro cultured HDMECs at passage 1 (P1) were stained for CD146, CD31 and Prox1. Two subpopulations of BECs (CD31^+^/ Prox1^−^) and LECs (CD31^+^/ Prox1^+^) are identified in HDMECs (panel I). CD146 expression is detected on a small fraction of CD31^+^ cells (panel II), while Prox1^+^cells lack the expression of CD146 (panel III). Triple immunofluorescence staining shows the expression of CD146 only in CD31^+^Prox1^−^ cells, corresponding to BECs (panel IV). Cell nuclei are stained with Hoechst (blue). Scale bar: 100 μm. (*n* = 3 independent donors). **C** Representative immunofluorescence images of BECs and LECs at passage 1 separated by FACS and cultured in vitro. CD31 and Prox1 staining confirm the purity of the two cell populations. Whereas CD146 expression is detected only on BECs, LECs lack the expression of CD146. Cell nuclei are stained with Hoechst (blue). Scale bar: 100 μm. (*n* = 3 independent donors). **D** Western blot analysis of sorted BECs and LECs shows the expression of CD146 only on BECs. Detection of Prox1 only on LECs confirmed the purity of the populations. Fibroblasts (FBs) were used as negative control. Equal loading was assessed with anti-GAPDH antibody. (*n* = 3 independent donors)

To perform a profound analysis of CD146 expression on endothelial cells, we performed complete digestions of the dermal fraction of human foreskins to isolate single cells. The single cell suspensions were then analyzed by flow cytometry for CD31, Podoplanin and CD146 expression (Fig. 4A). CD31 and podoplanin were used as markers to discriminate between blood endothelial cells (CD31^+^Podoplanin^-^) and lymphatic endothelial cells (CD31^+^ Podoplanin^+^). CD146 expression was then investigated among LECs and BECs gates. The results showed that CD146 expression was mainly detected on freshly isolated BECs (57,3 ± 7,4%), while almost no expression of CD146 was observed on freshly isolated LECs (2,6 ± 0,7%) (Fig. 4A). The detailed gating strategy is described in Supplementary Fig. S4A.

Further, we investigated the expression profile of CD146 on in vitro cultured HDMECs to verify if its expression could be also retained in vitro (Fig. 4B). Immunofluorescence staining for CD31 and the transcription factor Prox1 allowed us to discriminate between BECs (CD31^+^/ Prox1^−^) and LECs (CD31^+^/ Prox1^+^). As expected, CD31 stained all the endothelial cells, while Prox1 was detected only in a fraction of these cells, showing the presence of a mixed population of BECs and LECs in HDMECs (Fig. 4B panel I). Furthermore, we demonstrated that CD146 expression can be retained in vitro and in particular detected only in a population of HDMECs (Fig. 4B, panels II and III), which we showed to be negative for the lymphatic marker Prox1 (Fig. 4B, panel III). The triple co-immunofluorescence for CD31, CD146, and Prox1 conclusively indicated the expression of CD146 only on BECs (Fig. 4B, panel IV). We further validated our results on pure BECs and LECs separated by FACS (Supplementary Fig. S4B), to investigate CD146 expression on the single population without the interference of the other one (Fig. 4C). CD31 and Prox1 staining was performed to confirm the purity of the two cell populations (Fig. 4C). Whereas all the BECs resulted positive for CD146 expression, CD146 was not detected in LECs (Fig. 4C). These results were confirmed by western blot analysis, which displayed a specific band corresponding to the molecular weight of CD146 only in BECs (Fig. 4D). Prox1 protein expression was detected only in LECs, confirming the specificity of this marker. Meanwhile, CD31 expression was detected in both BECs and LECs, with lower expression levels in LECs (Fig. 4D), which is in agreement with a previous observation [23]. Fibroblasts (FBs) were used as negative control (Fig. 4D).

### Cultured CD146-positive pericytes displayed markers of perivascular cells

**Fig. 5:**
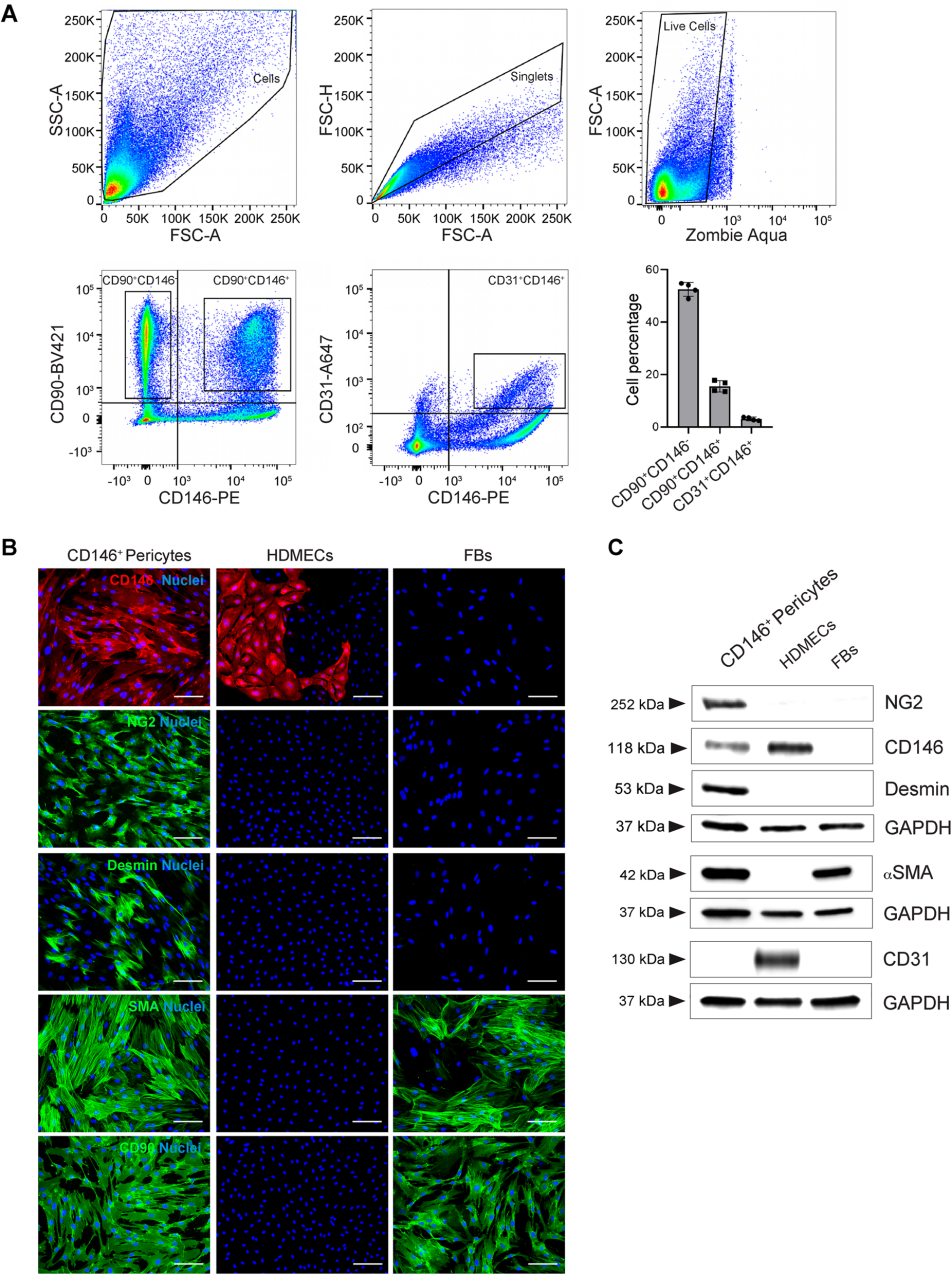
Characterization of CD146^+^ pericytes. **A** Flow cytometry analysis of a single cell suspension freshly isolated from human foreskin dermis. The hierarchical gating strategy involved the consecutive exclusion of debris, doublets, and dead cells using Zombie Aqua staining. The staining for CD146 and CD90 highlights the presence of two populations: pericytes (CD146^+^CD90^+^, 15,5 ± 2,1%) and fibroblasts (CD146^−^CD90^+^, 52,5 ± 2,6%). The staining with CD31 shows also the presence of a CD146^+^CD31^+^ cell population (3,1 ± 0,7%). *n* = 4 independent skin donors. **B** Representative immunofluorescence images of isolated CD146^+^ pericytes (*n *= 3 independent donors). The entire population of pericytes results positive for CD146, while HDMECs and FBs, used as control, displays a small population or no detection of CD146, respectively. Moreover, CD146^ +^ pericytes results entirely positive for NG2, while only a small percentage is also positive for desmin. CD146^ +^ pericytes are also positive for αSMA and CD90. No detection of these perivascular markers is observed on HDMECs, while FBs results positive for αSMA and CD90. Cell nuclei are stained with Hoechst (blue). Scale bar: 100 μm. **C** Western blot analysis of perivascular markers on CD146^ +^ pericytes, HDMECs and FBs. CD146^ + ^pericytes show protein expression of CD146, NG2, desmin, αSMA and CD90. HDMECs display higher protein expression of CD146 compared to pericytes. αSMA and CD90 are also detected on FBs. Equal loading was assessed with anti-GAPDH antibody. (*n* = 3 independent donors)

After our observations of CD146-positive pericytes surrounding blood capillaries in human skin (Fig. 3), we further characterized CD146 as a marker of human skin pericytes at single cell level. To this end, single cell suspensions freshly isolated from the human dermis were analyzed by flow cytometry (Fig. 5A) for CD146, CD90 and CD31 expression. Since CD90 represents a marker of both pericytes and fibroblasts, we could discriminate in our analysis of freshly isolated dermal cells two subpopulations attributable to pericytes (CD146^+^CD90^+^, 15,5 ± 2,1%) and fibroblasts (CD146^−^CD90^+^, 52,5 ± 2,6%) (Fig. 5A). A small population of CD146^ + ^CD31^ +^ cells (3,1 ± 0,7%) was gated among the entire live cell population (Fig. 5A). Thus, these results indicated that CD146 can be detected in both pericytes and endothelial cells. The detailed gating strategy is described in Supplementary Fig. S5.

Since CD146 has been described as a suitable marker for pericyte isolation, in particular in skeletal muscle and non-muscle tissues [10, 26], and our data on human skin sections showed that among the different perivascular markers CD146 is ﻿the most ubiquitously and strongly expressed, we decided to use CD146 as marker for human dermal pericyte isolation. Isolated CD146^+^ pericytes were then cultured in vitro and characterized for specific marker expression (Fig. 5B). First, we confirmed CD146 expression on all isolated pericytes by immunofluorescence (Fig. 5B). HDMECs, used as comparison, showed only a small fraction of CD146 positive cells, as expected, while FBs resulted negative. NG2 was uniformly expressed on all pericytes, while only a small fraction of cells was positive for desmin (Fig. 5B). This is in agreement with our observation on human skin sections that desmin positive pericytes represent only a small fraction of the entire pericyte population. CD146^+^ pericytes were also positive for αSMA and CD90 (Fig. 5B). HDMECs lacked the expression of these markers, while fibroblasts used as control were positive only for αSMA and CD90. Western blot analysis confirms these results (Fig. 5C). Interestingly, despite detectable CD146 expression in both pericytes and HDMECs, the protein levels were higher in HDMECs compared to pericytes (Fig. 5C).

### TNFα/IL-1β-mediated NFκB activation induces CD146 up-regulation in both pericytes and BECs

Next, we sought to investigate if the expression of CD146 could be modulated in pericytes and BECs by stimulation with the proinflammatory cytokines TNFα, IL-1β and IL-6.

To this aim, cells were stimulated with TNFα, IL-1β, or IL-6, for different time points and fluorescein diacetate–propidium iodide (FdA–PI) assay was performed to confirm that the treatments have not cytotoxic effect on cells (Supplementary Fig. S6).

**Fig. 6:**
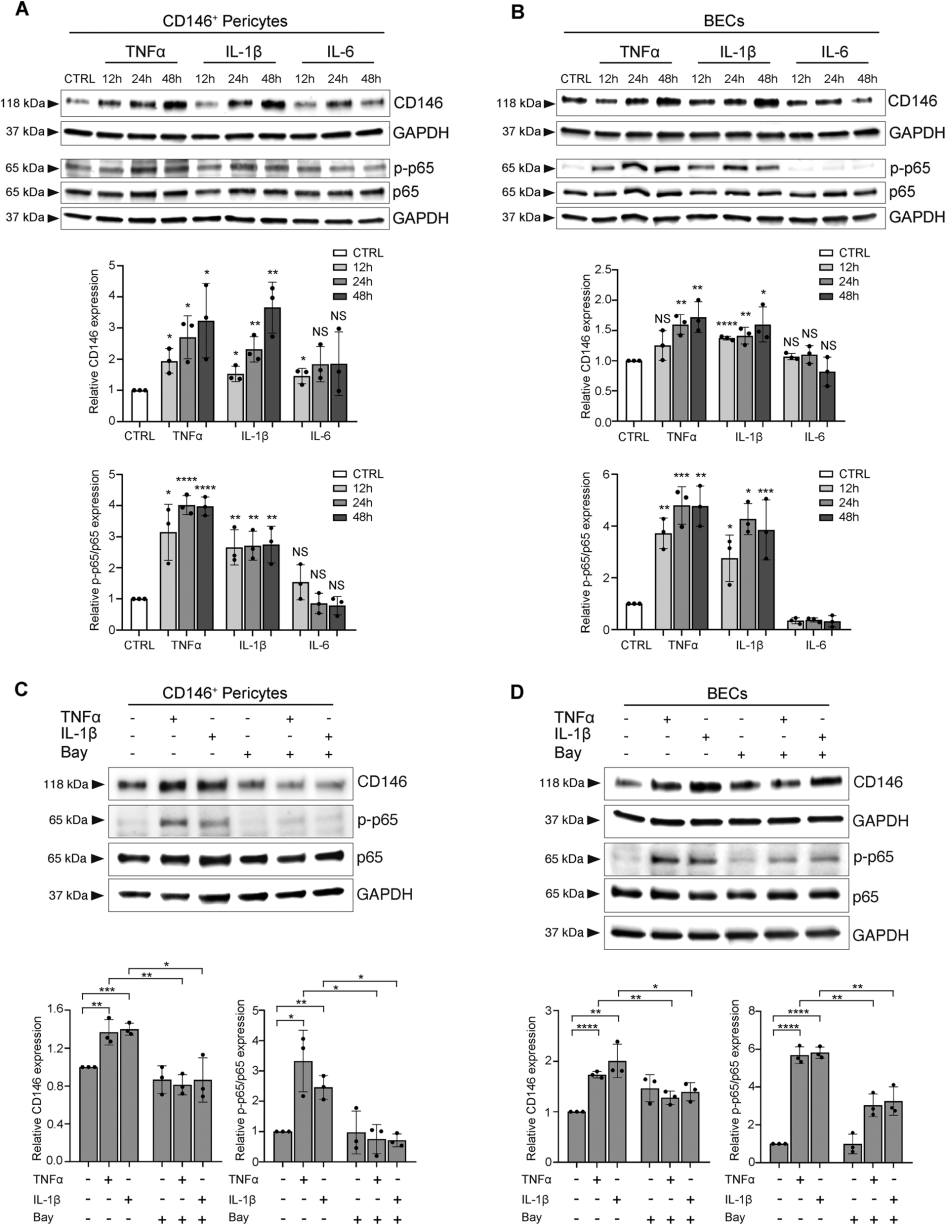
CD146 expression is up-regulated by stimulation with TNFα and IL-1β through NFκB activation.** A**, **B** CD146^+^ pericytes and BECs were left untreated or stimulated with TNFα, IL-1β, and IL-6 for 12 h, 24 h, and 48 h. Western blot analysis shows up-regulation of CD146 expression in response to TNFα and IL-1β treatment compared to unstimulated cells, while no changes in the protein levels of CD146 are observed after IL-6 stimulation. Parallel evaluation of p65 phosphorylation shows that only TNFα and IL-1β treatment induce p65 phosphorylation/activation in both pericytes and BECs. The equal loading was assessed using anti-GAPDH antibody for CD146 and anti-p65 for phospho-p65. ﻿For the densitometric analysis, the values from three independent experiments were normalized and expressed as fold increases and are reported as mean values ± standard deviations (SD). Unpaired Student’s t-test was performed, and significance levels are defined as * *p* < 0.05, ** *p* < 0.01, *** *p* < 0.001, and **** *p* < 0.0001. NS, not significant. **C,** **D **CD146.^+^ pericytes and BECs were left untreated or stimulated with TNFα or IL-1β in the presence or absence of the NFκB inhibitor, BAY 11–7082 (Bay) for 24 h. Western blot analysis shows that the increase in the levels of CD146 upon TNFα or IL-1β stimulation is abolished by treatment with NFκB inhibitor. The equal loading was assessed using anti-GAPDH antibody for CD146 and anti-p65 for phospho-p65. ﻿The densitometric analysis and unpaired Student’s t-test were performed as reported above. * *p* < 0.05, ** *p* < 0.01, *** *p* < 0.001, and **** *p* < 0.0001

Western blot analysis of CD146 expression showed a time-dependent up-regulation of CD146 protein levels after stimulation with TNFα or IL-1β in both pericytes (Fig. 6A) and BECs (Fig. 6B), while IL-6 treatment showed no effect on both pericytes and BECs (Fig. 6A and B). In addition, no induction of CD146 was observed in LECs stimulated with TNFα, IL-1β, or IL-6 (Supplementary Fig. S7).

Since both TNFα and IL-1β are known to activate NFκB [27], we investigated the activation of the NFκB pathway in pericytes and BECs. To this end, we analyzed the phosphorylation level of p65, one of the main NFκB subunits whose phosphorylation is required for full transcriptional activity of NFκB in the nucleus [28]. Both pericytes and BECs showed a significant phosphorylation/activation of p65 already after 12 h following stimulation with TNFα or IL-1β, which further increased after 24 h (Fig. 6A, B). IL-6 stimulation did not induce any phosphorylation/activation of p65/NFκB in both pericytes and BECs (Fig. 6A, B), as expected [27], thus strengthening the hypothesis that the regulation of CD146 proceeds through NFκB activation. To confirm the involvement of NFκB in the TNFα/IL-1β-mediated CD146 up-regulation, we stimulated the cells with the cytokines in the presence or absence of the specific NFκB inhibitor, BAY 11–7082 (Bay). The presence of the inhibitor significantly decreased the levels of CD146 induced by TNFα or IL-1β stimulation in both pericytes and BECs, which appeared to be more evident in pericytes (Fig. 6C, D). The down-regulation of the phosphorylation levels of p65 after Bay treatment, confirms the efficiency of the inhibitor (Fig. 6C, D). Thus, our results demonstrated that CD146 expression is up-regulated in both pericytes and BECs after stimulation with the pro- inflammatory cytokines TNFα and IL-1β through the activation of NFκB. 

### Engineering of 3D pre-vascularized hydrogel with HDMECs and CD146^+^ pericytes

**Fig. 7:**
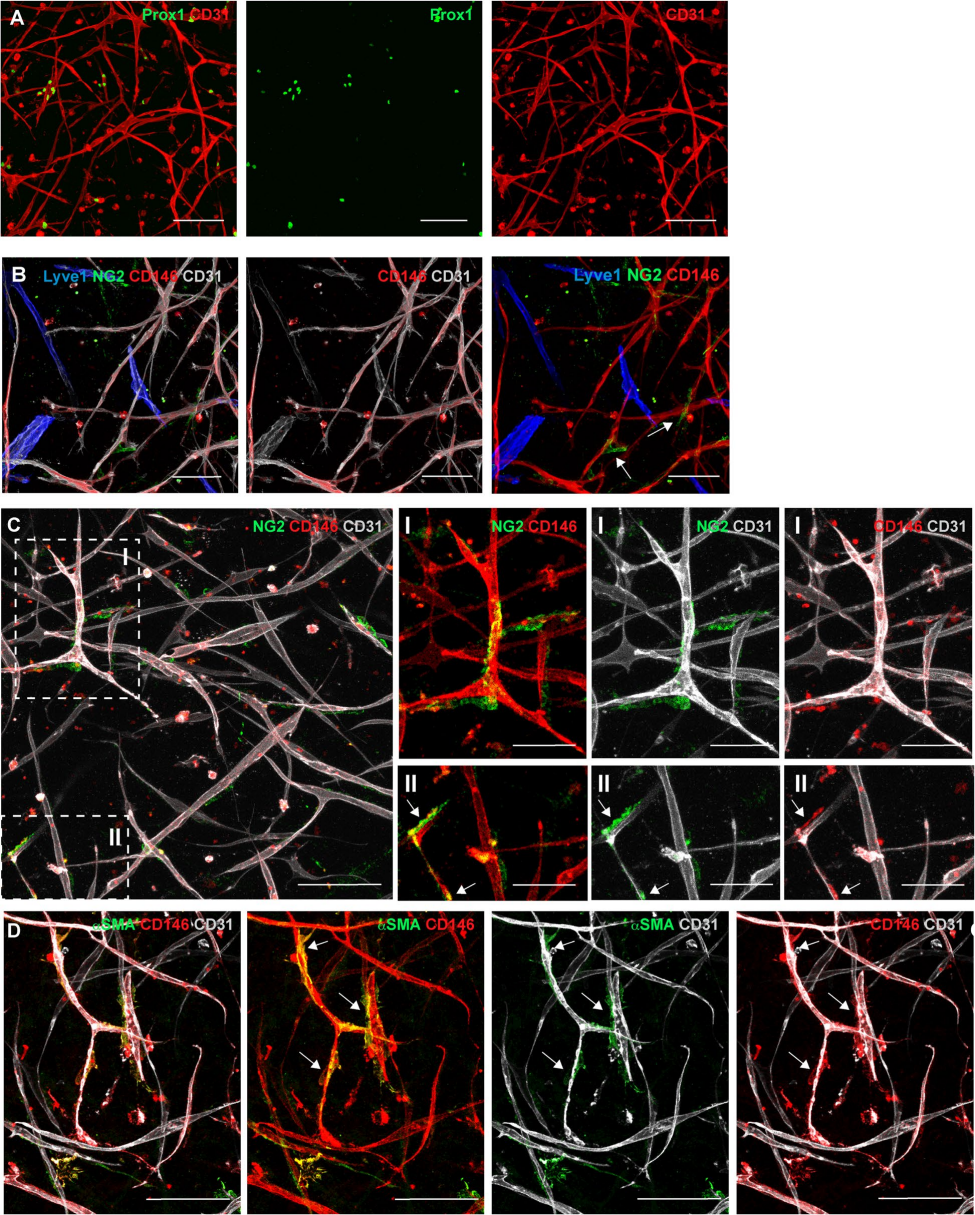
CD146 expression on 3D pre-vascularized hydrogel. Representative confocal images of HDMECs co-cultured with CD146^+^ pericytes and fibroblasts for three weeks in 3D collagen type I hydrogel. (*n* = 3 independent donors). **A** CD31 (red) and Prox1 (green) staining shows the developed vascular network composed of both blood and lymphatic capillaries. Scale bar: 100 μm. **B** Quadruple staining for CD146 (red), Lyve1 (blue), NG2 (green), and CD31 (white), shows that CD146 is expressed only on blood capillaries (CD31^+^Lyve1^−^), while lymphatic capillaries (CD31^+^Lyve1^+^) lack CD146 expression. Pericyte-like cells positive for NG2 are detected only around blood capillaries (CD31^+^Lyve1^−^) mimicking pericyte investment of the vessel and are not present on lymphatic capillaries. Scale bar: 100 μm. **C, D** Representative confocal images of CD146 and CD31 staining with NG2 (**C**) or αSMA (**D**) show the presence of pericytes-like cells double-positive for CD146^+^NG2^+^ (**C**, inset II, arrows) or CD146^+^αSMA^+^ (**D**, inset II, arrows), which surround blood capillaries. The presence of NG2-single positive cells is also observed around capillaries (**C**, inset I). Scale bar: 100 μm, inset 50 μm

We sought to reproduce a 3D model of vascularized dermis in vitro, in which the microvascular network resembled the human dermis microvasculature as closely as possible. To this end, we improved our well-established system of 3D pre-vascularized collagen type I hydrogels [21, 29], including also CD146^+^ pericytes. Whole-mount staining for CD31 and Prox1 in 3D hydrogels showed a developed vascular network, in which we could reproduce both blood capillaries and lymphatic capillaries (Fig. 7A). The specificity of CD146 for blood endothelial cells was conserved in our 3D hydrogels; in fact, CD146 expression was detected only on blood capillaries (CD31^+^/Lyve1^−^), while lymphatic capillaries (CD31^+^/Lyve1^+^) were CD146^−^ (Fig. 7B). NG2 staining displayed positive cells surrounding blood capillaries, mimicked pericyte investment of the vessel wall (Fig. 7B, C). As expected, lymphatic capillaries were missing these pericytes (Fig. 7B). Interestingly, in our 3D pre-vascularized hydrogels, we could observe the presence of NG2-single positive cells (Fig. 7B, C), as well as pericytes double-positive for CD146 and NG2 (Fig. 7C, panel II, arrows). The presence of single-positive cells for NG2 could be due to the loss of CD146 expression from some of these cells or to the differentiation of fibroblasts into pericytes. In fact, ﻿Goss and colleagues showed that mouse skin ﻿papillary and reticular fibroblasts can give rise to NG2^+^ pericytes in the upper and lower dermis respectively [30]. The whole-mount staining also showed the presence of CD31^ +^ blood capillaries surrounded by CD146^ + ^αSMA^ +^ (Fig. 7D, arrows), and CD146^ +^ CD90^ +^ (Supplementary Fig. S8) perivascular cells. Fibroblasts have been identified as single positive cells for CD90 (Supplementary Fig. S8).

### In vivo transplantation of pre-vascularized dermo-epidermal skin substitutes containing CD146^+^ pericytes

**Fig. 8:**
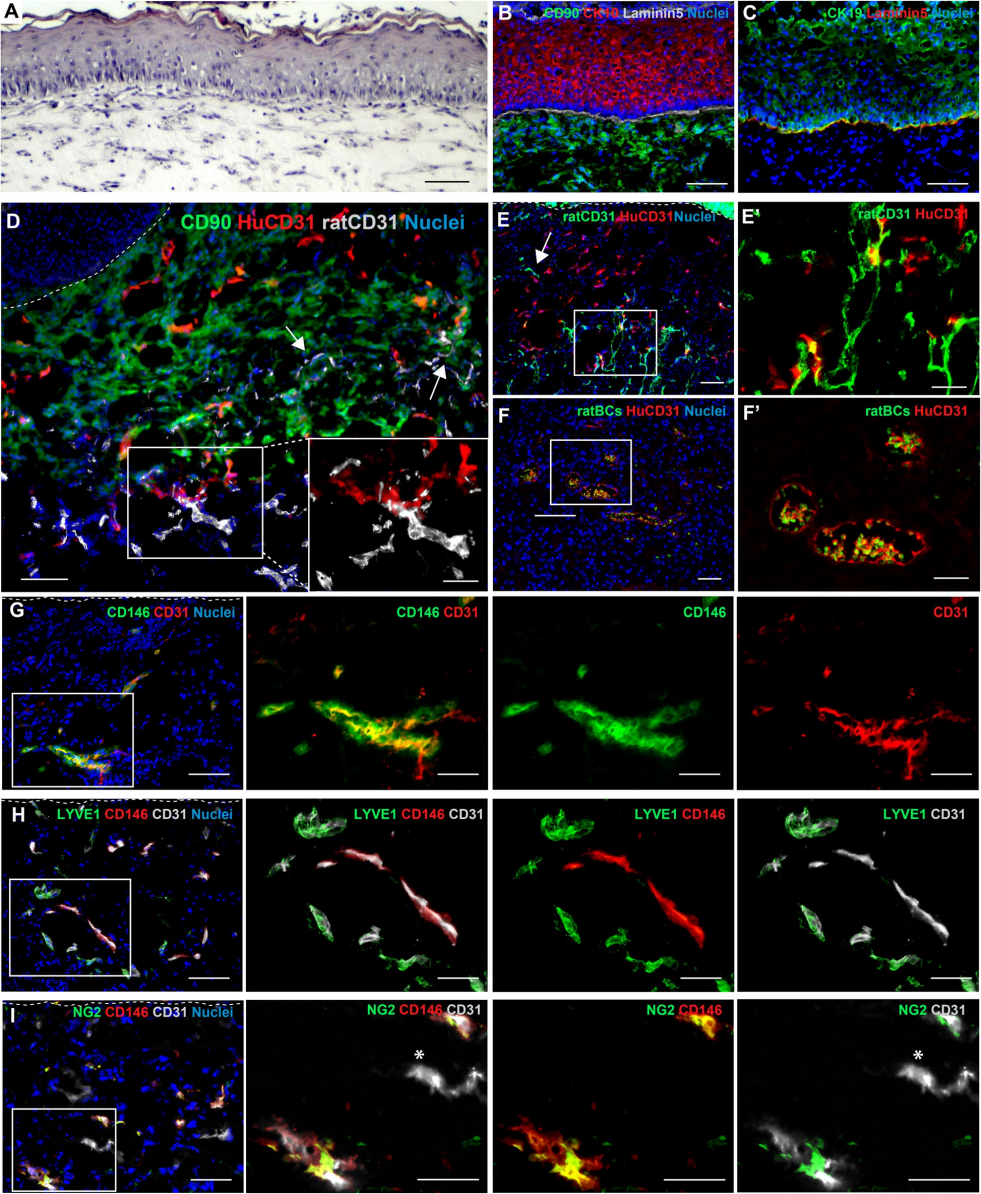
In vivo characterization of pre-vascularized DESS containing CD146^+^ pericytes. **A** The hematoxylin–eosin (H/E) staining of pre-vascularized DESS after 1 week shows a stratified epidermis and dermis containing fibroblasts. Scale bar: 100 μm. **B** The immunofluorescence staining for CK10 displays a positive signal of this keratinocyte differentiation marker from the suprabasal layer. Laminin332 and CD90 staining indicates the presence of basement membrane and human dermal fibroblasts, respectively. Scale bar: 100 μm. **C** The immunofluorescence staining for CK19 shows the presence of CK19-positive keratinocytes in both the basal and suprabasal layers. The staining for Laminin332 indicate the presence of the basement membrane. Scale bar: 100 μm. **D,** **E** Human capillaries positive for the specific human CD31 antibodies are distributed throughout the human dermis, which is marked with human CD90. Inosculation between human and rat capillaries is visualized by co-localization of humanCD31 and ratCD31 (**D, E**, insets). The ingrowth of rat capillaries is also observed in the human neo-dermis (arrows). White dashed lines indicate the dermo-epidermal junction. Scale bar: 100 μm, inset 50 μm. **F** The green autofluorescence of rat erythrocytes (ratBCs) inside the lumen of human capillaries indicates the presence of perfused capillaries. Scale bar: 100 μm, inset 50 μm. **G** Immunofluorescence staining for CD146 and humanCD31 shows the co-localization between these two markers and the presence of human CD146^+^ pericytes around capillaries. Scale bar: 100 μm, inset 50 μm. **H** Lymphatic capillaries stained for Lyve1 are CD146 negative. **I** Human pericytes double-positive for CD146 and NG2 are observed around capillaries. CD146 negative capillaries, attributable to lymphatic capillaries, show no presence of pericytes (asterisk). **G**, **H**, **I** (*n* = 3 independent donors). White dashed lines indicate the epidermal-dermal junction. Scale bar: 100 μm, inset 50 μm

The 3D pre-vascularized hydrogels composed of endothelial cells, CD146^+^ pericytes, and fibroblasts were then covered with human keratinocytes (KC) to generate pre-vascularized dermo-epidermal skin substitutes (DESS) and transplanted onto the back of immuno-incompetent rats to investigate whether they might be able to form a stable and functional vascular network in vivo. Histological analysis of the skin substitutes collected after one week showed the presence of a stratified epidermis composed of stratum basale and several suprabasal layers until the stratum corneum, as well as a dermal compartment containing fibroblasts (Fig. 8A). The corresponding immunofluorescence analysis showed the expression of the differentiation marker cytokeratin 10 (CK10) throughout the suprabasal layers, while the basal layer displayed no expression of CK10. The staining for Laminin332 showed the presence of a basement membrane between the epidermis and the human dermis, which has been visualized with the antibody against human CD90 (Fig. 8B). Evaluation of epidermis homeostasis has been performed with the analysis of cytokeratin 19 (CK19) expression, which is usually confined to the stratum basale of the epidermis in engineered skin substitutes after three weeks of transplantation [20]. Our results showed that after one week from skin graft transplantation, CK19 was expressed not only in the cells of the basal layer, but also in the suprabasal layers of the epidermis, indicating that epidermis homeostasis had not yet reached (Fig. 8C).

To investigate the presence of the human vascular network in the skin substitutes and its ability to anastomose with the host vascular plexus, we performed immunofluorescence analysis with antibodies specifically discriminating between CD31 of human and rat origin (Fig. 8D, E). CD90 staining was performed to distinguish between the human dermis and the rat tissue (Fig. 8D). An extensive network of human blood capillaries was distributed throughout the human dermis (Fig. 8D, E). Here, the presence of ratCD31-single positive capillaries indicated the ingrowth of the host blood vessels into the human skin transplant to support ﻿blood perfusion (Fig. 8D, E, arrows). The colocalization between human capillaries and rat capillaries, particularly observed at the interface between human and rat tissues, indicated inosculation between the two microvascular networks (Fig. 8D, E insets). In addition, rat red blood cells (ratBCs) were found in the lumen of several capillaries lined by human endothelial cells (Fig. 8F, inset). All these data provided evidence for the functionality of the transplanted human microvascular network and its efficiency in blood circulation.

Further, we assessed the expression of CD146 in blood capillaries and pericytes in the pre-vascularized transplants. We found the presence of human capillaries double-positive for humanCD31 and CD146, surrounded by perivascular cells single-positive for CD146 (Fig. 8G, Supplementary Fig. S9A). Moreover, lymphatic capillaries stained with Lyve1 or Prox1 displayed negative expression of CD146 (Fig. 8H, Supplementary Fig. S9B), confirming the exclusive expression of CD146 on blood capillaries. CD146/NG2 double-positive human pericytes were also found investing CD146-CD31 positive capillaries (Fig. 8I, Supplementary Fig. S9C), while CD31 single-positive capillaries attributable to lymphatic capillaries showed no presence of pericytes around (Fig. 8I, asterisk), confirming the role of pericytes in stabilizing only the blood capillaries.

The immunofluorescence staining for CD146, humanCD31 and ratCD31 showed a co-localization of CD146 only with capillaries from human origin (Supplementary Fig. S9D), confirming the specificity of the CD146 antibody for the human tissue and excluding the possibility that the the observed CD146-positive pericyte could have been of rat origin.

## Discussion

In this study, we characterized CD146 as a specific marker of blood endothelial cells and pericytes in human skin in vitro and in vivo*,* whose expression is up-regulated by stimulation with the pro-inflammatory cytokine TNF-α and IL-1β. In addition, herein we report a method to isolate and culture human CD146^+^ pericytes in order to combine them with HDMECs and fibroblasts in 3D pre-vascularized skin substitutes, to improve and stabilize the vascular network.

First, we focused on the expression and characterization of CD146 in human skin as marker of both endothelial cells and pericytes. Alternative co-staining of CD146 with pan-endothelial CD31 and markers specific for blood endothelial cells (PLVAP) or lymphatic endothelial cells (Lyve1, podoplanin and Prox1) conclusively identify CD146 as a specific marker of blood endothelial cells both in vitro and in vivo. Interestingly, the expression of CD146 is retained in BECs after isolation and in vitro culturing, allowing us to track its expression from the in situ skin biopsies to the 2D cultures and finally 3D hydrogels. These results are in line with a previous study showing the different gene profile expression of BECs and LECs [18]. Thus, our data contribute to identifying CD146 as a specific marker that effectively discriminates between blood and lymphatic endothelial cells. In fact, most of the markers used to identify endothelial cells, like CD31, CD34, and VE-cadherin, cannot really discriminate between blood and lymphatic endothelial cells, while several studies are mainly focused to identify specific markers of lymphatic cells [18, 31, 32].

We also observed a fraction of CD146^+^CD31^−^ cells surrounding the blood vessels. The co-staining of CD146 with different markers of perivascular cells confirms that CD146 is also expressed on pericytes. In particular, CD146^+^ pericytes were detected almost within the entire blood microcirculatory network, colocalizing with NG2, while only a small fraction of CD146^+^desmin^+^ cells﻿ was detected in the lower dermis. In addition, the co-localization between CD146 and αSMA, which is a marker of vascular smooth muscle cells (VSMCs), showed that CD146 is expressed not only in pericytes surrounding small capillaries, but also in VSMCs [33]. Thus, our results suggest the presence of heterogeneous populations of pericytes displaying different marker expression, in which CD146 seems to be the most significantly expressed.

For this reason, we chose CD146 as an eminently suitable marker to isolate and culture in vitro pericytes from human skin. We observed that the isolated CD146^+^ pericytes conserved in vitro the expression of the other perivascular markers previously analyzed in human skin sections. This is in line with another study showing that isolated pericytes from human placental tissue expressed the typical pericyte markers NG2, CD90, CD146, αSMA, and PDGFRβ, and further, that the pericytes can be easily expanded in vitro [34].

CD146 is also known to be up-regulated during inflammation, where it is involved in the recruitment and transmigration of mononuclear cells across the endothelium [35], suggesting an important role of CD146 as mediator of the inflammatory process. However, CD146 expression needs to be spatiotemporally regulated, in order to increase during inflammation to stimulate angiogenesis, and then decrease again. Indeed, excessive overexpression of CD146 has been correlated with several chronic inflammatory diseases [35, 36]. For example, in multiple sclerosis CD146 plays a crucial role in mediating lymphocyte transmigration across the blood–brain barrier and inducing neuronal inflammation [37]. In systemic sclerosis patients show high levels of different isoforms of soluble CD146 (scCD146), which are responsible for profibrotic effects through dysregulation of the Wnt-1/ β-catenin pathway and proangiogenic activity via up-regulation of the PKCε pathway [36]. Moreover, aberrant up-regulation of CD146 has been associated with various cancers, where it is involved in the early step of cancer formation and progression through vascular metastasis [1, 14, 35].

Skin wound healing is a complex process that involves different consecutive phases: homeostasis, inflammation, proliferation, and remodeling, which requires a coordinated interplay between different cell types, cytokines, chemokines, and growth factors [38, 39]. Thus, we speculated that CD146 expression could be modulated during this process, in particular during the inflammatory phase, to support vascularization at the wound site. Consistent with this hypothesis, we found that CD146 was up-regulated by the stimulation with the pro-inflammatory cytokine TNFα and IL-1β in both pericytes and BECs. In parallel, we also observed the activation of the NF-κB pathway, while the stimulation with IL-6 did not induce neither CD146 up-regulation nor NF-κB activation, suggesting a correlation between these two events. Indeed, the use of the specific NF-κB inhibitor could revert the up-regulation of CD146 observed upon TNFα and IL-1β treatments, confirming that CD146 expression is induced by TNFα and IL-1β through NF-κB activation. Interestingly, we observed a stronger effect of the NF-κB inhibitor on p65 phosphorylation on pericytes compared to BECs, which consequently led to a more evident down-regulation of CD146 expression. These differences could be explained by a different susceptibility of pericytes and BECs to the inhibitor. Moreover, since the stimulation with TNFα or IL-1β induced a higher p65 phosphorylation in BECs compared to pericytes, the treatment with NF-κB inhibitor might be less efficient in down-regulating CD146 expression in BECs. Our results are in agreement with previous findings of other groups showing the involvement TNFα-IL-1β/NF-κB pathway in the regulation of CD146 expression in HUVECs [15, 16]. Moreover, previous studies showed that the up-regulation of CD146 induced by pro-inflammatory cytokines is required for the recruitment and trans-endothelial migration of monocytes during inflammation [16, 40].

Further investigations would be necessary to assess if CD146 could play an important role in both pericytes and BECs during the inflammatory phase of skin wound healing to allow not only angiogenesis but also trans-endothelial migration of monocytes.

The engineering of autologous full-thickness skin graft represents one of the most promising approaches for the treatment of full-thickness skin defects, and a lot of effort has been directed to the realization of dermo-epidermal skin substitutes (DESS) [41–43]. However, one of the main issues related to this approach is the insufficient initial vascularization, which may result in a lack of nutrients and oxygen after transplantation, with consequent impaired regeneration [44]. For this reason, the engineering of pre-vascularized 3D tissues represents an innovative approach to allow better engraftment of the new tissue [44–46]. Recently, we developed a 3D system of pre-vascularized DESS (vascDESS) composed of both blood and lymphatic capillaries, obtained from an in vitro system of co-cultured HDMECs and human dermal fibroblasts. These vascDESS showed a rapid in vivo perfusion after transplantation [21, 29, 47, 48]. However, to improve the survival and integration of large engineered skin, it is crucial to mimic all the complexity of human microvasculature as much as possible, thus incorporating also pericytes [49–51].

The presence of pericytes is crucial to stabilize and improve the functionality of the vascular network. In our previous works, we observed human capillaries surrounded by perivascular cells of rat origin in vivo [29, 52], meaning that a stable vascular network needed the presence of pericytes, which had to migrate from the vascular bed of the host to the human capillaries.

We previously demonstrated that pre-vascularized skin grafts engineered with the stromal vascular fraction (SVF) of human adipose tissue generated a functional vascular network composed of blood capillaries and pericytes which allowed better tissue engraftment (30). Indeed, SVF consists of a heterogeneous cell population including endothelial cells, pericytes, and other cell types like adipose stromal cells and multipotent stem cells with high vasculogenic potential (31, 32). In addition, few studies show a role of pericytes in improving epithelial proliferation and epidermal regeneration in skin tissue engineering [53, 54]. Thus, our interest was focused on studying and characterizing human skin pericytes, and incorporating them into our pre-vascularized DESS engineered with HDMECs and fibroblasts.

Pericytes are perivascular cells surroundings endothelial cells lining capillaries, microvessels, and large vessels, usually not expressed on lymphatic vessels. These cells play a crucial role in vascular development, stabilization, maturation, and remodeling. In addition, they control blood pressure and permeability of blood vessels [55–57]. It has been shown that the presence of pericytes can stabilize microvessels both in vivo and in vitro [34, 58, 59].

Herein, we managed to reproduce for the first time 3D pre-vascularized dermo-epidermal skin grafts composed of HDMECs and CD146^+^ pericytes in vitro and in vivo. We developed a microvascular network in which CD146 was exclusively expressed on blood capillaries and not detected on lymphatic capillaries, confirming our observation in vivo and in 2D cultures. In addition, we observed pericytes double-positive for CD146 and the other perivascular markers NG2, αSMA, and CD90 exclusively investing blood capillaries while no presence of pericytes on lymphatic capillaries was detected [60]. The realization of a pre-existing vascular plexus already composed of both human pericytes and blood capillaries could accelerate and improve the engraftment of the tissue.

Moreover, we showed that the combination of pericytes and HDMECs in 3D hydrogels was able to produce a functional vascular network, which is perfused and connected with ﻿the blood capillaries of the host. This condition is fundamental to ensure tissue engraftment and skin regeneration.

Our results are also interesting in comparison to our previously published works regarding pre-vascularized DESS containing only fibroblasts and HDMECs. In fact, here we showed that already after 1 week post-transplantation, we obtained an extensive and perfused vascular network comparable to what was observed after 3 weeks post-transplantation in DESS without pericytes [59, 61]. Moreover, in the skin substitutes engineered without pericytes, capillaries start to be stabilized by mural cells only after 3/4 weeks in vivo, probably the time necessary for fibroblast differentiation or rat pericyte recruitment on the capillaries [52, 59]. Here, we observed the entire blood capillary network stabilize by CD146^ +^ pericytes after 1 week. For a more comprehensive study, future investigations should require a direct comparison of skin substitutes engineered with or without pericytes in long-term in vivo studies.

However, the entire study was conducted only on cells isolated from the juvenile foreskin, which is an excellent source for isolating large quantities of cells with a high proliferation rate, unlike other adult skin biopsies. Nevertheless, cells isolated from different anatomical sites can exhibit great variability. For example, fibroblasts derived from different body sites show heterogeneity which is then reflected in different wound healing and fibrotic responses [62]. Furthermore, single-cell analysis revealed endothelial cell heterogeneity among different human organs/tissues as well as within different skin tissues, such as limbs, trunk, and foreskin [63]. We have previously shown that mesenchymal cells derived from different body sites can support tissue-engineered DESS maturation in vivo [64], as well as endothelial cells isolated from ﻿adipose stromal vascular fraction (SVF) also support skin substitutes vascularization [29], thus representing possible alternative sources for future clinical applications. Future research may also focus on studying CD146 expression and pericyte characterization in cells isolated from different skin sites.

Further functional studies will be necessary to investigate the role of CD146 in pericytes-endothelial cells interaction and its contribution during wound healing in dermo-epidermal skin substitutes in vivo.

## Supplementary Information

**Additional file 1: Supplementary Fig. S1. **PLVAP expression on human blood capillaries. **A**, **B**, **C**, and **D** Representative immunofluorescence images of human skin sections co-stained for PLVAP with CD31, or with the markers of lymphatic endothelial cells, Lyve1, Podoplanin, Prox1. PLVAP co-localize with CD31 (**A**), but it is not expressed on lymphatic vessels (**B**, **C**, **D**). Scale bar:50 μm. **E** Individual channels of the immunofluorescence stainings shown in Figure 1E for human skin sections stained for CD146, PLVAP, CD31. (*n* = 3 independent donors). Scale bar: 50 μm. **Supplementary Fig. S2.** Individual channels of immunofluorescence stainings in Figure 2. **A**, **B**, and **C** Individual channels of the immunofluorescence stainings shown in Figure 2 for human skin sections stained for CD146, CD31 and Podoplanin (**A**) or Lyve1 (**B**) or Prox1 (**C**). (*n* = 3 independent donors). Scale bar: 50 μm. **Supplementary Fig. S3.** Individual channels of immunofluorescence stainings in Figure 3. **A**, **B**, and **C** Individual channels of the immunofluorescence stainings shown in Figure 3 for human skin sections stained for CD146, CD31 and NG2 (**A**) or desmin (**B**) or αSMA (**C**). The dotted line represents the dermo-epidermal junction. (*n* = 3 independent donors). Scale bar: 50 μm. **Supplementary Fig. S4.** Gating strategy for flow cytometric analysis of freshly isolated HDMECs from human foreskin dermis. **A** The gates have been established based on isotype controls, unstained and single stained cells. Fluorescence-minus-one (FMO) controls were performed to defined the positive gates. **B** Hierarchical gating strategy performed to separates BECs and LECs. Gates were settled to consecutive exclude debris, doublets, and dead cells. Staining with CD31-PE and Podoplanin-A488 allowed to sort HDMECs into BECS (CD31^+^Podoplanin^-^) and LECs (CD31^+^Podoplanin^+^). The two cell populations have been reanalyzed by flow cytometry to confirm their purity. (*n* = 3 independent donors). **Supplementary Fig. S5.** Gating strategy for flow cytometric analysis of freshly isolated cells from human dermis. The gates have been established based on unstained and single stained cells. Fluorescence-minusone (FMO) controls were performed to define the positive gates. (*n* = 3 independent donors). **Supplemental Fig. S6.** Cell viability of pericytes and BECs after stimulation with cytokines. **A,** **B** CD146^+^ pericytes and BECs were left untreated or stimulated with TNFα, IL-1β and IL-6 for 12h, 24h and 48h. Live/dead staining with fluorescein diacetate–propidium iodide (FdA–PI, FdA in green, PI in red) was performed at the indicated time points to assess cell viability. Scale bar: 100 μm. (*n*=3). **Supplementary Fig. S7.** CD146 expression does not appear in LECs stimulated with proinflammatory cytokines. LECs were left untreated or stimulated with TNFα, IL-1β, and IL-6 for 12h, 24h, and 48h. Western blot analysis shows no detection of CD146 expression in both control condition and after stimulation with TNFα, IL-1β, and IL-6. Unstimulated BECs were used as positive control for CD146 expression. The equal loading was assessed using anti-GAPDH. (*n* = 3 independent donors). **Supplementary Fig. S8.** CD146/CD90 expression on cells in a 3D-prevascularized hydrogel. Cells in 3D collagen type I hydrogel are stained for CD146, CD90 and CD31. Representative confocal images show CD146/CD90-double positive pericyte-like cells surrounding blood capillaries. Single CD90-positive cells identify fibroblasts. (*n* = 3 independent donors). Scale bar: 100 μm, inset 50 μm. **Supplemental Fig. S9.** CD146 expression in prevascularized dermo-epidermal skin substitutes in vivo. **A,** **B** CD146 expression is detected on CD31-positive capillaries and on pericytes-like cells investing these capillaries (**A**), but CD146 is not present on Prox1-positive lymphatic capillaries (**B**). White dashed lines indicate the dermo-epidermal junction. Scale bar 100 μm, inset 50 μm. **C** CD146 colocalizes with NG2 in human pericytes associated to CD31-positive capillaries. Scale bar: 100 μm, inset 50 μm. **D** The co-localization between CD146 and humanCD31 and the absence of CD146 expression on rat capillaries confirm the specificity of the CD146 antibody for human capillaries. (*n* = 3 independent donors). Scale bar: 100 μm, inset 50 μm. Dotted line represents dermo-epidermal junction. **Supplementary Table 1.** List of antibodies The following primary and secondary antibodies were used for immunofluorescence (IF), whole mount (WM), western blot (WB), FACS (FC).

## Data Availability

The datasets used and/or analyzed during the current study are available from the corresponding author upon reasonable request.

## Abbreviations

BECs: Blood endothelial cells
BSA: Bovine serum albumin
CK10: Cytokeratin 10
CK19: Cytokeratin 19
DESS: Dermo-epidermal skin substitutes
FB: Human dermal fibroblasts
FDA: Fluorescein diacetate
HDMECs: Human dermal microvascular endothelial cells
HUVECs: Human umbilical vein endothelial cells
KC: Human keratinocytes
LECs: Lymphatic endothelial cells
MCAM: Melanoma cell adhesion molecule
PI: Propidium iodide
PLVAP: Plasmalemma vesicle-associated protein
ratBCs: Rat red blood cells
SD: Standard deviation
VSMCs: Vascular smooth muscle cells
