# Supplementary material for: CD146 expression profile in human skin and pre-vascularized dermo-epidermal skin substitutes in vivo
Source: J Biol Eng. 2023 Jan 31;17:9. doi: 10.1186/s13036-023-00327-x (PMC9890844; doi:10.1186/s13036-023-00327-x)
Supplement: Supplementary file 1 — Additional file 1: Supplementary Fig. S1. PLVAP expression on human blood capillaries. A, B, C, and D Representative immunofluorescence images of human skin sections co-stained for PLVAP with CD31, or with the markers of lymphatic endothelial cells, Lyve1, Podoplanin, Prox1. PLVAP co-localize with CD31 (A), but it is not expressed on lymphatic vessels (B, C, D). Scale bar:50 μm. E Individual channels of the immunofluorescence stainings shown in Figure 1E for human skin sections stained for CD146, PLVAP, CD31. (n = 3 independent donors). Scale bar: 50 μm. Supplementary Fig. S2. Individual channels of immunofluorescence stainings in Figure 2. A, B, and C Individual channels of the immunofluorescence stainings shown in Figure 2 for human skin sections stained for CD146, CD31 and Podoplanin (A) or Lyve1 (B) or Prox1 (C). (n = 3 independent donors). Scale bar: 50 μm. Supplementary Fig. S3. Individual channels of immunofluorescence stainings in Figure 3. A, B, and C Individual channels of the immunofluorescence stainings shown in Figure 3 for human skin sections stained for CD146, CD31 and NG2 (A) or desmin (B) or αSMA (C). The dotted line represents the dermo-epidermal junction. (n = 3 independent donors). Scale bar: 50 μm. Supplementary Fig. S4. Gating strategy for flow cytometric analysis of freshly isolated HDMECs from human foreskin dermis. A The gates have been established based on isotype controls, unstained and single stained cells. Fluorescence-minus-one (FMO) controls were performed to defined the positive gates. B Hierarchical gating strategy performed to separates BECs and LECs. Gates were settled to consecutive exclude debris, doublets, and dead cells. Staining with CD31-PE and Podoplanin-A488 allowed to sort HDMECs into BECS (CD31+Podoplanin-) and LECs (CD31+Podoplanin+). The two cell populations have been reanalyzed by flow cytometry to confirm their purity. (n = 3 independent donors). Supplementary Fig. S5. Gating strategy for flow cytometric analysis of f [file 13036_2023_327_MOESM1_ESM.pdf]

Supplementary Figures

CD146 expression profile in human skin and pre-vascularized dermo-epidermal skin substitutes *in vivo*

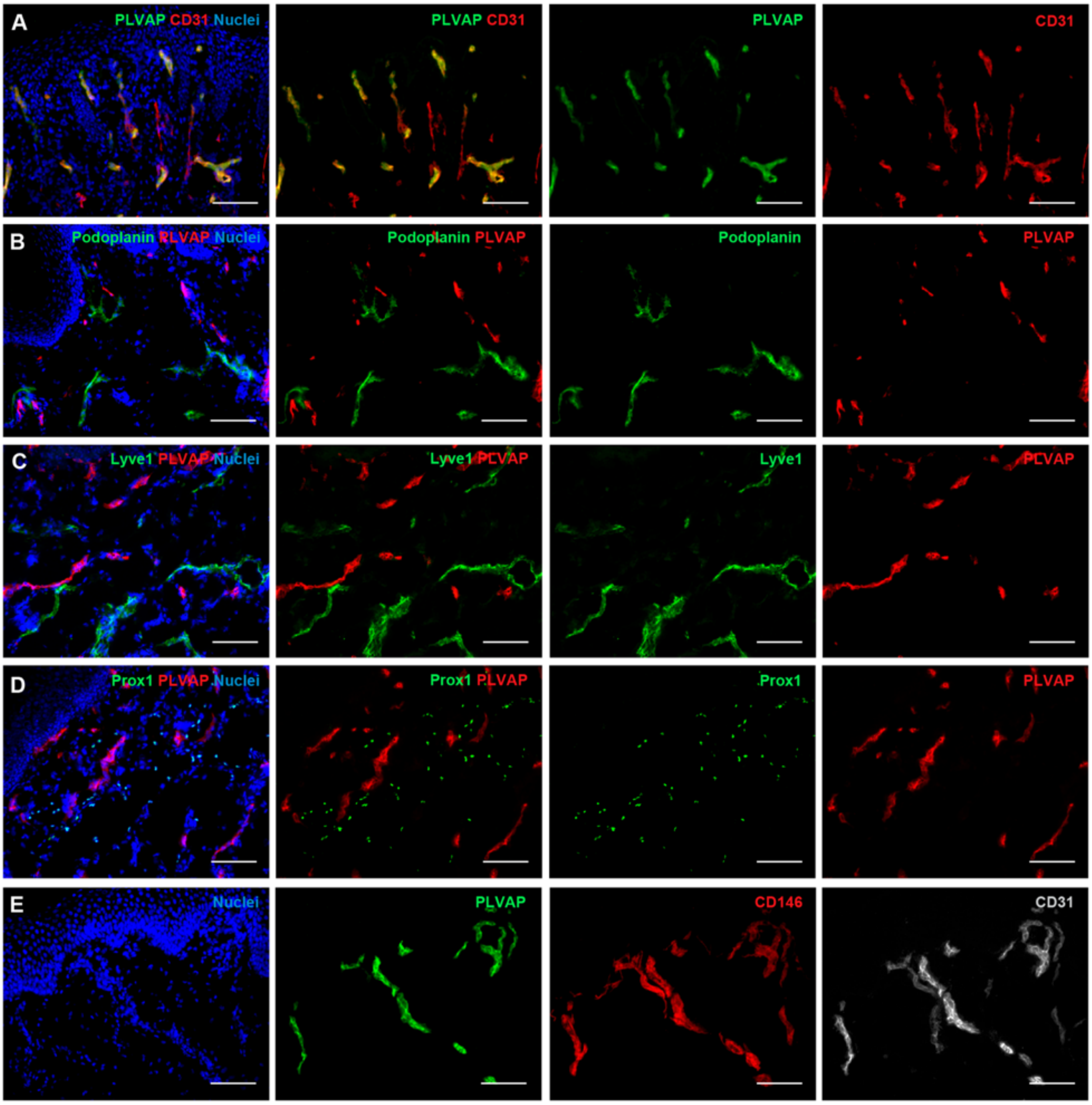

Supplementary Fig. S1. PLVAP expression on human blood capillaries.

(**A**, **B**, **C**, and **D**) Representative immunofluorescence images of human skin sections co-stained for PLVAP with CD31, or with the markers of lymphatic endothelial cells, Lyve1, Podoplanin, Prox1. PLVAP co-localize with CD31 (**A**), but it is not expressed on lymphatic vessels (**B, C, D**). Scale bar:50 µm. (**E**) Individual channels of the immunofluorescence stainings shown in Figure 1E for

human skin sections stained for CD146, PLVAP, CD31. (n = 3 independent donors). Scale bar: 50 µm.

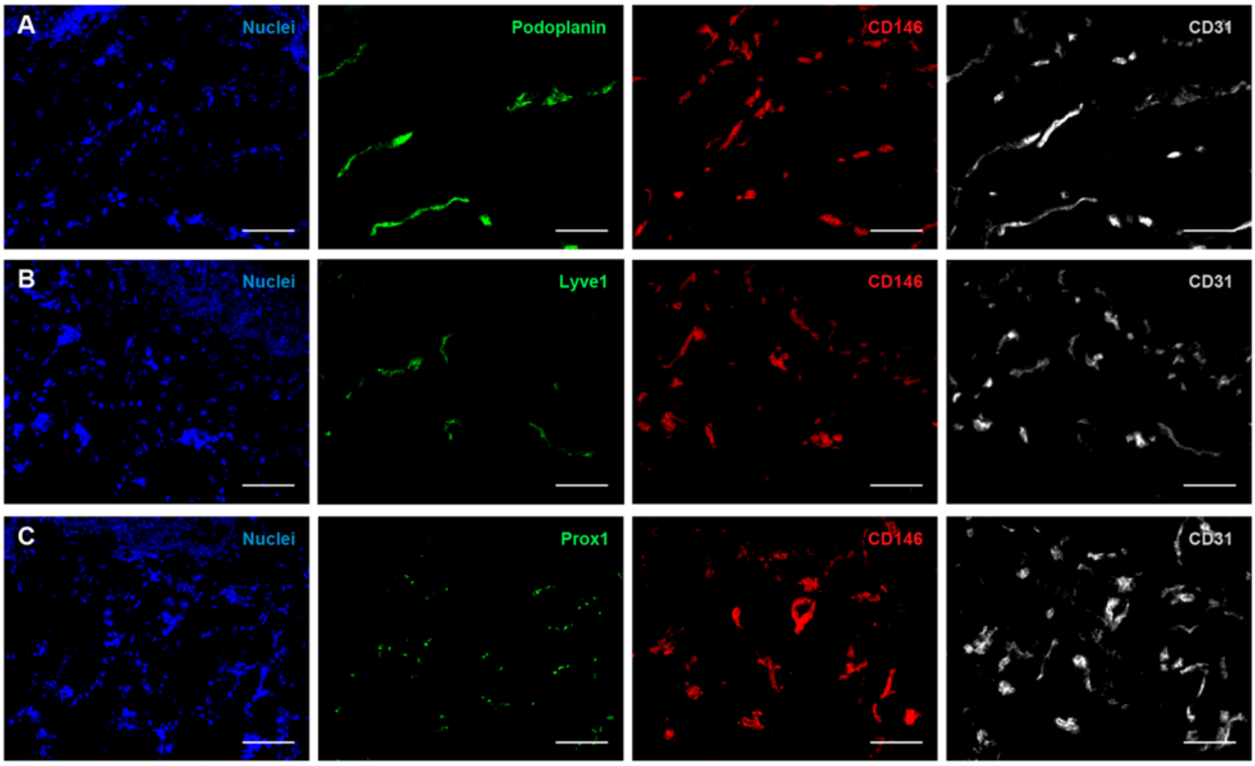

Supplementary Fig. S2. Individual channels of immunofluorescence stainings in Figure 2. (**A**, **B**, and **C**) Individual channels of the immunofluorescence stainings shown in Figure 2 for human skin sections stained for CD146, CD31 and Podoplanin (**A**) or Lyve1 (**B**) or Prox1 (**C**). (n = 3 independent donors). Scale bar: 50 µm.

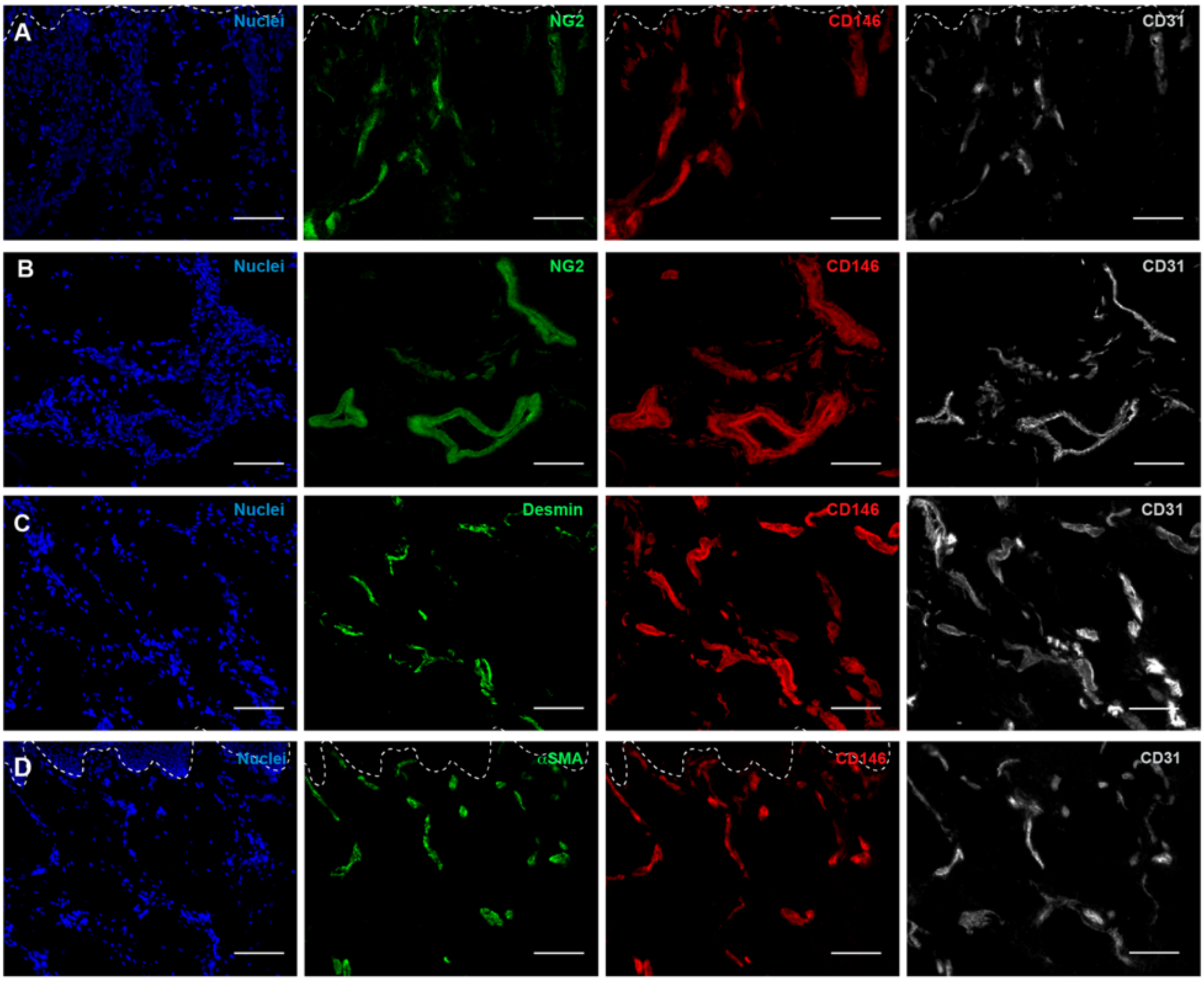

Supplementary Fig. S3. Individual channels of immunofluorescence stainings in Figure 3. **(A**, **B**, and **C)** Individual channels of the immunofluorescence stainings shown in Figure 3 for human skin sections stained for CD146, CD31 and NG2 (**A**) or desmin (**B**) or αSMA (**C**). The dotted line represents the dermo-epidermal junction. (n = 3 independent donors). Scale bar: 50 μm.

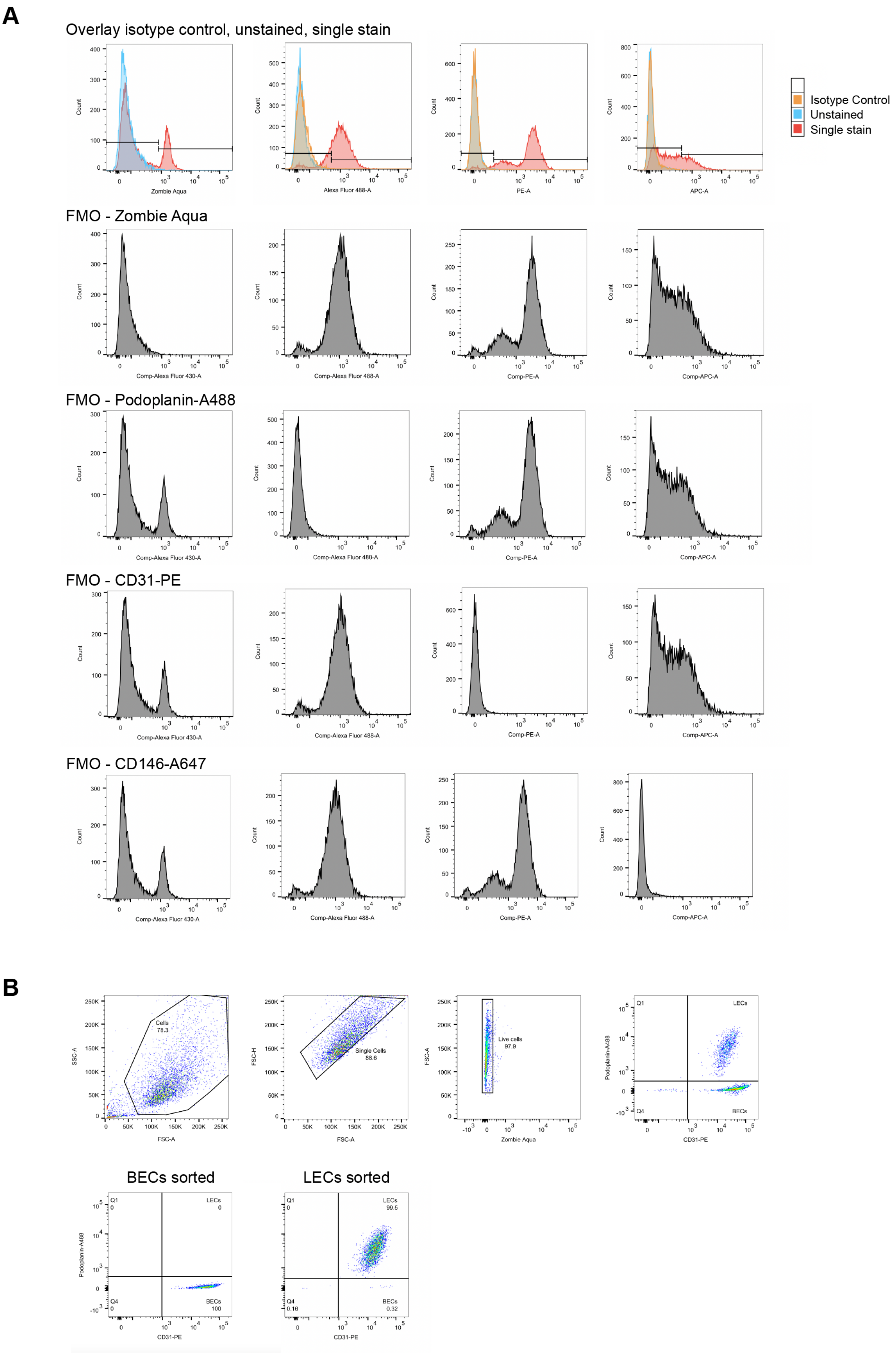
A
Overlay isotype control, unstained, single stain
Isotype Control
Unstained
Single stain
Zombie Aqua
Alexa Fluor 488-A
PE-A
APC-A
FMO - Zombie Aqua
FMO - Podoplanin-A488
FMO - CD31-PE
FMO - CD146-A647
Comp-Alexa Fluor 430-A
Comp-Alexa Fluor 488-A
Comp-PE-A
Comp-APC-A
Count
B
Cells
78.3
Single Cells
88.6
Live cells
97.9
SSC-A
FSC-A
FSC-H
Zombie Aqua
Podoplanin-A488
CD31-PE
Q1
LECs
Q4
BECs
BECs sorted
LECs sorted
Q1
0
LECs
0
Q4
0
BECs
100
Q1
0
LECs
99.5
Q4
0.18
BECs
0.32

Supplementary Fig. S4. Gating strategy for flow cytometric analysis of freshly isolated HDMECs from human foreskin dermis.

(**A**) The gates have been established based on isotype controls, unstained and single stained cells. Fluorescence-minus-one (FMO) controls were performed to defined the positive gates. (**B**) Hierarchical gating strategy performed to separates BECs and LECs. Gates were settled to consecutive exclude debris, doublets, and dead cells. Staining with CD31-PE and Podoplanin-A488 allowed to sort HDMECs into BECS ($CD31^+Podoplanin^-$) and LECs ($CD31^+Podoplanin^+$). The two cell populations have been reanalyzed by flow cytometry to confirm their purity. ($n = 3$ independent donors).

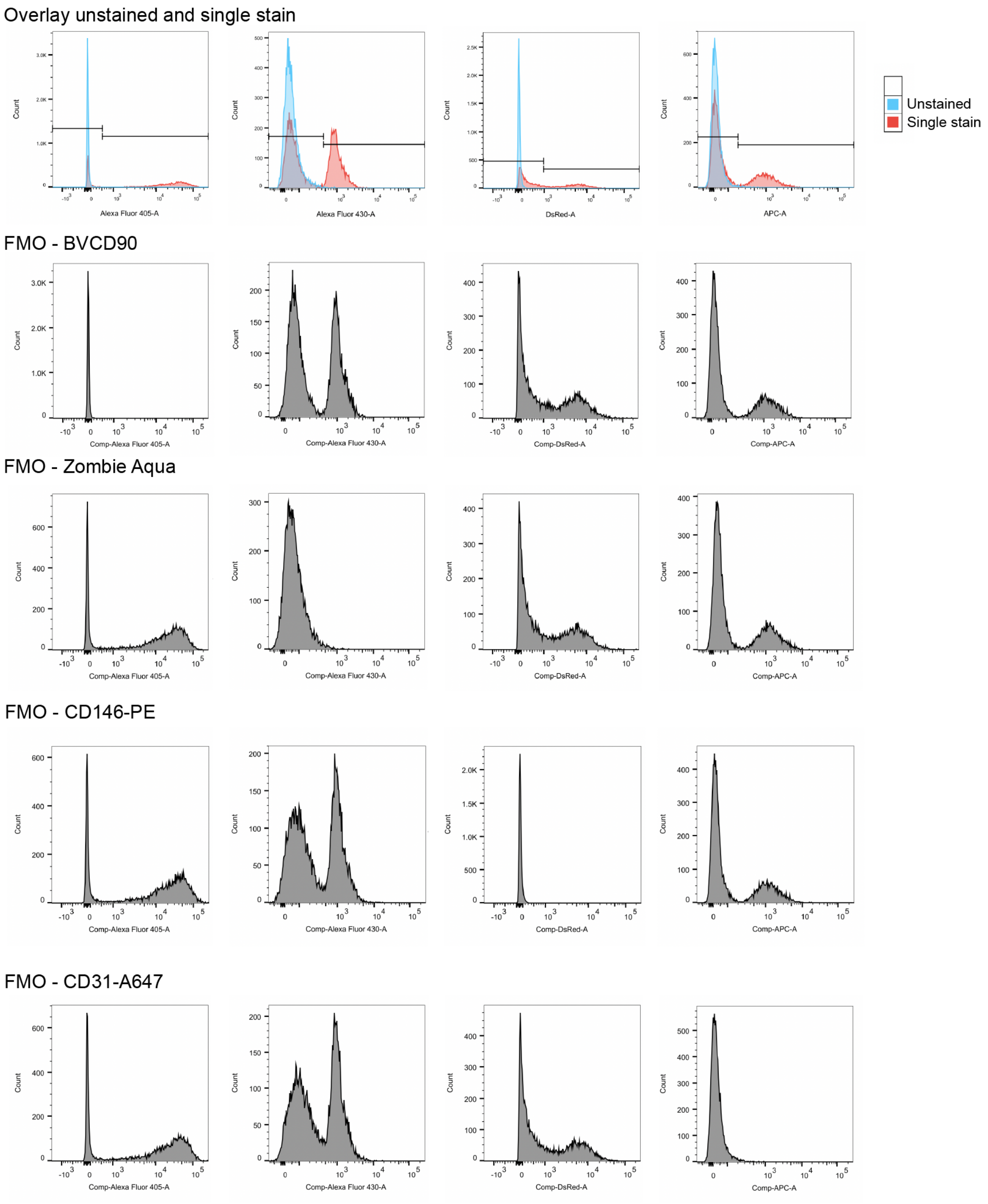

Supplementary Fig. S5. Gating strategy for flow cytometric analysis of freshly isolated cells from human dermis.

The gates have been established based on unstained and single stained cells. Fluorescence-minus-one (FMO) controls were performed to define the positive gates. (n = 3 independent donors).

A

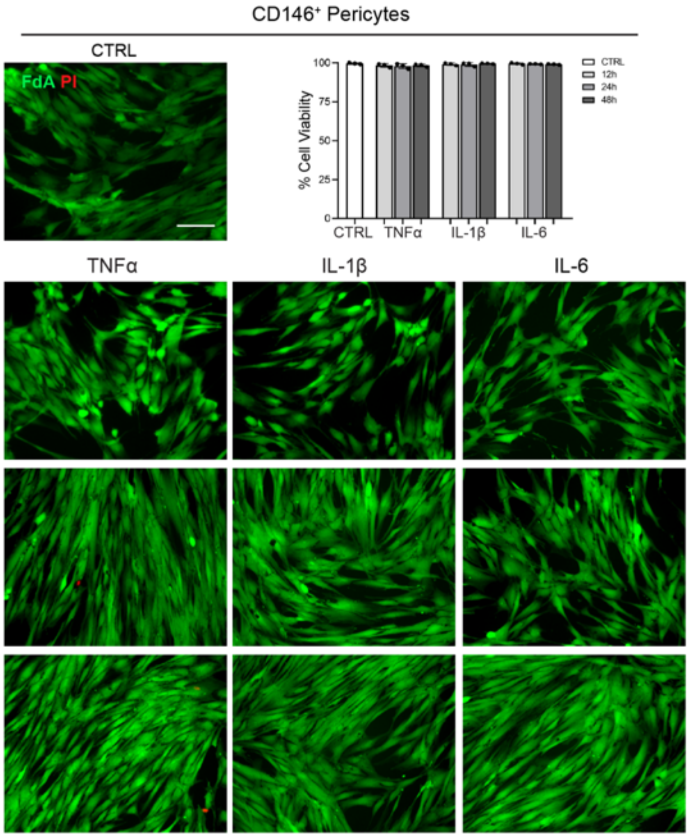
CD146+ Pericytes
CTRL
FdA PI
% Cell Viability
0
25
50
75
100
CTRL
12h
24h
48h
CTRL TNFα IL-1β IL-6
TNFα
IL-1β
IL-6
12h
24h
48h

B

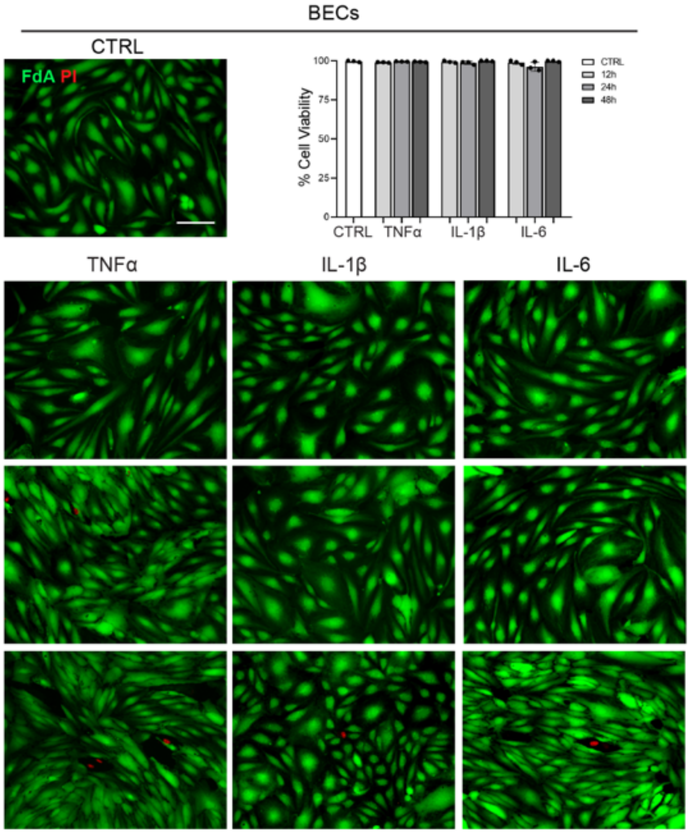
BECs
CTRL
FdA PI
% Cell Viability
0
25
50
75
100
CTRL
12h
24h
48h
CTRL TNFα IL-1β IL-6
TNFα
IL-1β
IL-6
12h
24h
48h

Supplementary Fig. S6. Cell viability of pericytes and BECs after stimulation with cytokines. (**A** and **B**) CD146$^+$ pericytes and BECs were left untreated or stimulated with TNFα, IL-1β and IL-6 for 12h, 24h and 48h. Live/dead staining with fluorescein diacetate–propidium iodide (FdA–PI, FdA in green, PI in red) was performed at the indicated time points to assess cell viability. Scale bar: 100 µm. (n=3).

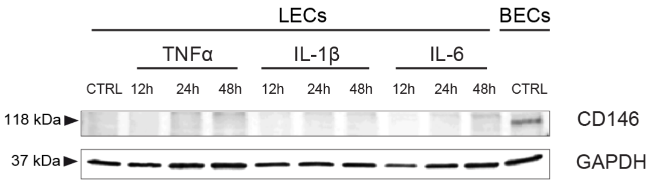

Supplementary Fig. S7. CD146 expression does not appear in LECs stimulated with pro-inflammatory cytokines. LECs were left untreated or stimulated with TNFα, IL-1β, and IL-6 for 12h, 24h, and 48h. Western blot analysis shows no detection of CD146 expression in both control condition and after stimulation with TNFα, IL-1β, and IL-6. Unstimulated BECs were used as positive control for CD146 expression. The equal loading was assessed using anti-GAPDH. (n = 3 independent donors).

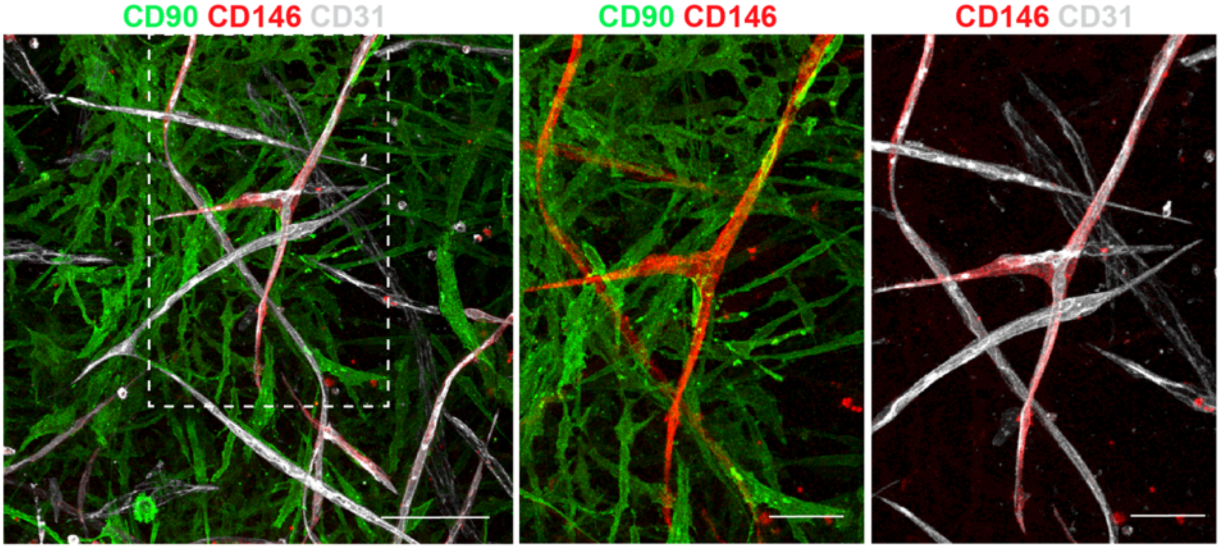

Supplementary Fig. S8. CD146/CD90 expression on cells in a 3D-prevascularized hydrogel. Cells in 3D collagen type I hydrogel are stained for CD146, CD90 and CD31. Representative confocal images show CD146/CD90-double positive pericyte-like cells surrounding blood capillaries. Single CD90-positive cells identify fibroblasts. (n = 3 independent donors). Scale bar: 100 µm, inset 50 µm.

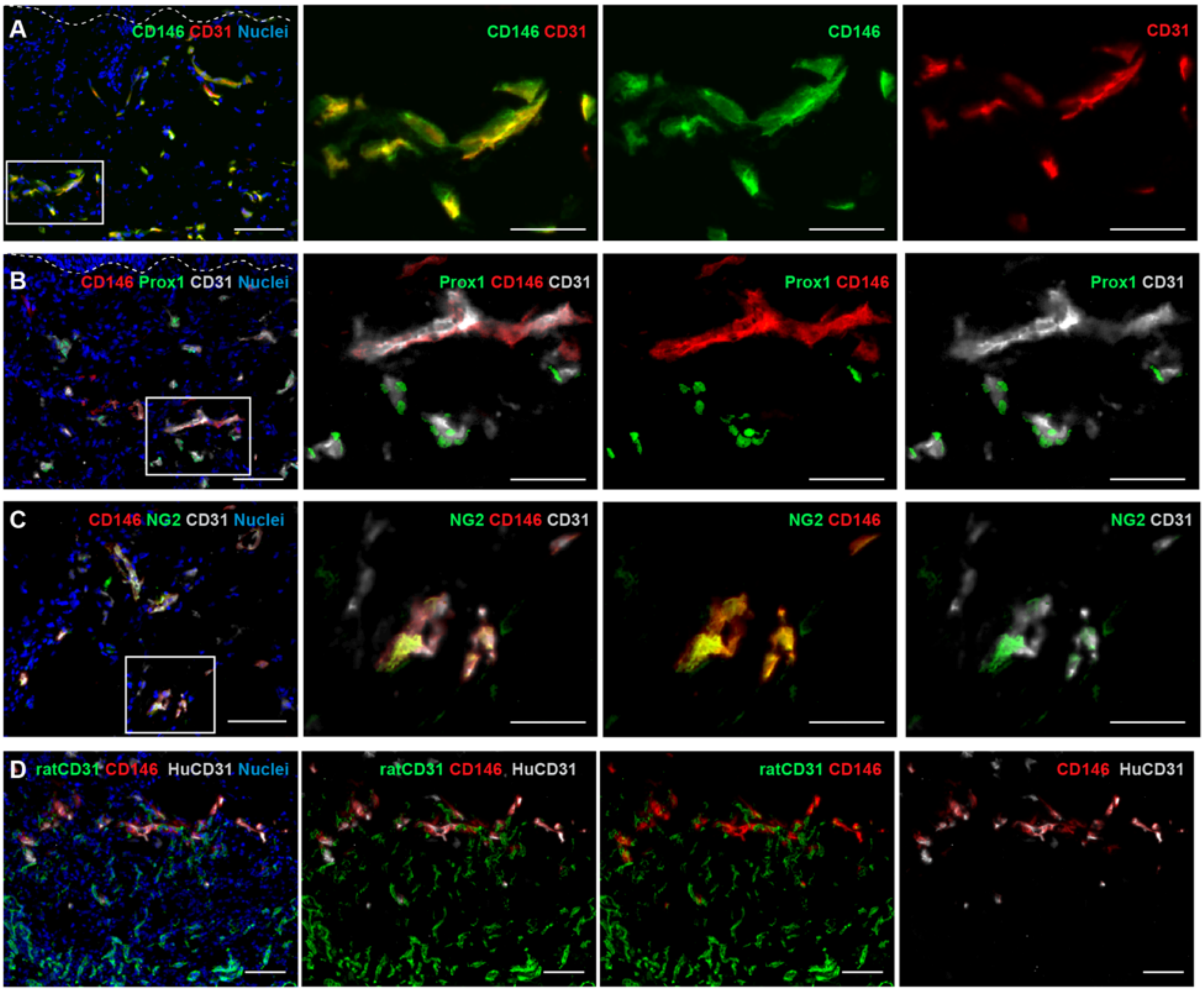

Supplementary Fig. S9. CD146 expression in prevascularized dermo-epidermal skin substitutes *in vivo*.

(**A** and **B**) CD146 expression is detected on CD31-positive capillaries and on pericytes-like cells investing these capillaries (**A**), but CD146 is not present on Prox1-positive lymphatic capillaries (**B**). White dashed lines indicate the dermo-epidermal junction. Scale bar 100 µm, inset 50 µm. (**C**) CD146 colocalizes with NG2 in human pericytes associated to CD31-positive capillaries. Scale bar: 100 µm, inset 50 µm. (**D**) The co-localization between CD146 and humanCD31 and the absence of CD146 expression on rat capillaries confirm the specificity of the CD146 antibody for human capillaries. (n = 3 independent donors). Scale bar: 100 µm, inset 50 µm. Dotted line represents dermo-epidermal junction.

Supplementary Table 1. List of antibodies

The following primary and secondary antibodies were used for immunofluorescence (IF), whole mount (WM), western blot (WB), FACS (FC).

Antibody	Clone	Company	Catalog number	Dilution
Anti-human CD146	P1H12	Biolegend	361002	IF 1:50, WB:1:500
Anti-human CD146-PE	P1H12	Biolegend	361005	IF 1:50, WM 1:50
Anti-human CD31	JC70A	Dako	M083	IF 1:50, WM 1:50, WB 1:1000
Anti-human CD31-647	JC70A	Abcam	ab215912	IF 1:100, WM 1:100
Anti-human CD31-PE	WM-59	BD Bioscience	555446	IF 1:50, FC 1:20
Anti-human PLVAP	174/2	Abcam	ab81719	IF 1:50
Anti-human Podoplanin-488	NC-08	Biolegend	337006	IF 1:20, FC 1:40
Anti-human Lyve1		Abcam	ab10278	IF 1:50, WM 1:100
Anti-human Prox1		ReliaTech	102-PA32AG	IF 1:100, WM 1:100, WB 1:1000
Anti-human α-SMA	1A4	Dako	M0851	IF 1:100, WB 1:5000
Anti-human Desmin		Abcam	ab8592	IF 1:50, WB 1:1000
Anti-human NG2	LHM 2	Novus Biologicals	NB100-2688	IF 1:50, WM 1:50, WB 1:500
Anti-human NG2-488	9.2.27	BD Bioscience	562413	WM 1:50
Anti-human CD90-FITC	5E10	Biolegend	328108	IF 1:20
Anti-human CD90-BV421	5E10	Biolegend	328121	IF 1:100
Anti-human Laminin5		Abcam	ab14509	IF 1:100
Anti-human CK10	DE-K10	Dako	M7002	IF 1:100
Anti-human CK19		Abcam	ab52625	IF 1:100
Anti-human NFκB p65	A-12	Santa Cruz Biotechnology	sc-514451	WB 1:1000
Anti-human NFκB p65 (phospho)		Abcam	ab86299	WB 1:2000
Anti-rat CD31		Abcam	ab222783	IF 1:100
Anti-human GAPDH	6C5	Millipore	MAB374	WB 1:5000
**Secondary Antibodies**				
donkey anti-mouse IgG H&L Alexa Fluor A488		Abcam	ab150105	IF 1:400, WM 1:400
donkey F(ab')2 anti-mouse IgG H&L Alexa Fluor A568		Abcam	ab175699	IF 1:400, WM 1:400
donkey anti-rabbit IgG H&L Alexa Fluor A488		Abcam	ab150073	IF 1:400
goat anti-mouse IgG H&L Alexa Fluor A647		Abcam	ab150115	IF 1:200
donkey anti-rabbit IgG H&L Alexa Fluor A405 preadsorbed		Abcam	ab175649	IF 1:200
Goat Anti-Mouse Immunoglobulins/HRP		Dako	P0447	WB 1:3000
Swine Anti-Rabbit Immunoglobulins/HRP		Dako	P0399	WB 1:3000
